# Polyethylene glycol and proline synergistically improve salinity tolerance via physiological and biochemical reprogramming in mango

**DOI:** 10.1186/s12870-025-07211-4

**Published:** 2025-08-29

**Authors:** Mohamed El Kheshin, Ibrahim Hmmam

**Affiliations:** Pomology Department, Faculty of Agriculture, Cairo University, PO box 12613, Giza, Egypt

**Keywords:** Abiotic stress, Antioxidant enzymes, Hormonal regulation, *Mangifera indica*, Osmotic adjustment, PEG, Proline, Salinity

## Abstract

**Background:**

Mango (*Mangifera indica* L.) is a globally important fruit crop, but its sensitivity to salt stress poses a serious threat to its sustainable cultivation. Salt stress impairs mango growth through osmotic imbalance, ion toxicity, oxidative damage, and reduced nutrient uptake. This study examined the biochemical, physiological, vegetative, and reproductive responses of the Egyptian mango cultivar ‘Ewais’ under constant salinity stress reflecting the naturally saline irrigation water in the orchard. The research specifically aimed to evaluate the efficacy of polyethylene glycol (PEG) and proline in mitigating the adverse effects of salt exposure. Nine treatments were tested over two consecutive growing seasons (2023 and 2024). The treatments consisted of PEG applied individually at 15 mM (T_1_) and 30 mM (T_2_), as well as proline applied at 8 mM (T_3_) and 13 mM (T_4_). Four combinations were also examined, including T_1_ + T_3_ (T_5_), T_2_ + T_4_ (T_6_), T_1_ + T_4_ (T_7_), and T_2_ + T_3_ (T_8_). A control group (T_9_) consisted of trees subjected to the same salinity conditions without any application of PEG or proline.

**Results:**

The treatments investigated revealed significant improvements in relative water content (RWC), membrane stability index (MSI), peroxidase (POD) and polyphenol oxidase (PPO) activities, chlorophyll concentration, ascorbate accumulation, proline concentration, total soluble sugar metabolism, and the hormonal balance of indole-3-acetic acid (IAA) and abscisic acid (ABA). Also, positive responses were observed in vegetative growth traits such as shoot elongation, number of flushes, and leaf area, as well as in reproductive traits including fruit set percentage, average fruit weight, and yield. Specifically, the combined treatments of PEG and proline, T_6_ (PEG 30 mM + Proline 13 mM) and T_8_ (PEG 30 mM + Proline 8 mM), led to sustained improvements in physiological and agronomic performance.

**Conclusion:**

The findings support a dual-action mechanism in which PEG triggers osmotic signalling, while proline contributes to maintaining antioxidant defences and metabolic stability, thereby establishing PEG-proline co-application as a promising strategy for enhancing mango productivity under saline conditions.

**Supplementary Information:**

The online version contains supplementary material available at 10.1186/s12870-025-07211-4.

## Introduction

Mango (*Mangifera indica* L.) holds a distinguished place in global agriculture due to its rich nutritional profile, cultural symbolism, and robust international trade presence. Native to South Asia but now cultivated across tropical and subtropical regions worldwide, mango plays a pivotal role in the livelihoods of millions of smallholder farmers, particularly in countries like India, Mexico, Thailand, Brazil, and Egypt [1]. In Egypt, mango cultivation is a key component of the horticultural sector, supporting rural employment and contributing to both local markets and export revenues. Its diverse varieties cater to a broad range of consumer preferences, making it integral to food security and rural economies. However, mango cultivation faces numerous challenges, including vulnerability to pests, inconsistent flowering, and sensitivity to climatic fluctuations. Among these challenges, salinity stress is one of the most critical abiotic factors affecting mango production, particularly in arid and semi-arid regions. Mango, a commercially valuable tropical fruit tree, is moderately sensitive to salt stress, which interferes with its physiological and biochemical functions. This interference results in reduced vegetative growth, impaired flowering, and lower fruit yield [2–5]. In Egypt, salinity is a major obstacle to agricultural development, especially in the reclamation of new lands where saline irrigation water is common. The high salt concentration in these areas restricts the expansion of mango cultivation and limits the sustainable production of this economically important fruit crop.

It is also worth noting that salinity represents a major obstacle to the implementation of the Sustainable Development Strategy and Egypt’s Vision 2030. Salinity induces osmotic stress, ion toxicity, and oxidative damage that compromise water balance, membrane integrity, and photosynthetic efficiency. Salinity poses complex and detrimental effects on fruit crops, notably restricting their growth and productivity. In mango and similar species, high salinity levels have been observed to reduce shoot and root elongation, decrease leaf production, and lower overall biomass, all of which contribute to visibly stunted development. These growth limitations are often accompanied by declines in fruit yield and quality, along with abnormalities in floral structures. Salinity also interferes with photosynthetic efficiency by lowering chlorophyll and carotenoid levels, which in turn compromises the energy production necessary for plant development [2]. Furthermore, saline conditions impair water relations within the plant, reducing its ability to absorb moisture and leading to internal water deficits [3, 6]. Excessive salt disrupts ionic balance by hindering the uptake of essential nutrients like potassium and calcium, while encouraging the accumulation of sodium ions. On a cellular scale, salinity-induced oxidative stress causes damage to vital structures, further weakening plant vitality [6]. To counteract these effects, plants employ complex protective mechanisms, including osmotic adjustment through proline buildup, osmolyte accumulation, enhanced antioxidant defence, and hormonal rebalancing, all of which contribute to maintaining physiological stability under saline conditions [7–9]. Salinity stress progressively disrupts key physiological functions critical to mango productivity. It initially impairs nutrient uptake, undermining root performance and diminishing leaf turgor, both of which are essential for optimal photosynthetic activity. As photosynthesis declines, carbohydrate synthesis is reduced, which in turn delays floral initiation and compromises flower viability. As a result, pollen fertility and fruit set decrease, ultimately leading to a reduction in final yield [10, 11]. Each stage in this cascade is interconnected and essential, highlighting how early disruptions under salinity stress can progressively impact overall crop performance. Numerous studies have explored the application of various compounds and technologies to alleviate the detrimental effects of salinity stress on mango trees. These include the use of ascorbic acid [12], nanotechnology-based interventions [13], plant growth regulators [14, 15], and silicon-based treatments [16, 17]. Among these, ascorbic acid plays a key role by enhancing nutrient uptake and activating antioxidant enzymes, which help mitigate oxidative stress [12]. Through these mechanisms, it strengthens plant resilience and supports sustained growth under saline conditions.

Exogenous application of osmotic agents such as polyethylene glycol (PEG) and compatible solutes like proline offers a promising approach to enhance plant resilience through physiological priming and metabolic buffering [18–25]. PEG, a non-ionic polymer, induces water deficit stress without contributing to ionic toxicity, thereby stimulating plant osmotic adjustment mechanisms including ABA accumulation and aquaporin activation. Proline, an endogenous osmolyte, functions as a molecular chaperone, ROS scavenger, and membrane stabilizer, helping maintain cellular homeostasis under salinity [26–30]. Despite existing studies on the separate influence of PEG and proline in some perennial fruit species, their combined effects on mango trees have not been adequately addressed and remain relatively unexplored.

Consequently, PEG and proline have been widely used in plant stress physiology due to their distinct roles in enhancing tolerance to environmental stressors. PEG serves as a non-penetrating osmotic agent that mimics drought-like conditions, reducing water potential and helping plants adjust osmotically without causing toxicity. Proline, a naturally occurring amino acid, contributes to osmotic adjustment, membrane stabilization, and the scavenging of reactive oxygen species. The combined use of PEG and proline is of particular interest for its potential to enhance stress tolerance through complementary physiological mechanisms. To the best of our knowledge, this is the first report to assess this specific treatment combination under saline conditions in mango, offering novel insights into integrated stress management strategies for woody horticultural species.

Therefore, the present study was conducted to evaluate the individual and combined effects of PEG and proline on mature mango trees (cv. ‘Ewais’) under salinity stress. The research focused on a wide range of parameters, including physiological, biochemical, hormonal, vegetative, and reproductive traits, assessed over two consecutive seasons. The goal was to better understand the mechanisms underlying PEG–proline interaction in stress mitigation and to inform practical strategies for improving mango performance in saline environments.

## Materials and methods

The experiment was conducted over two consecutive growing seasons, in 2023 and 2024, on 54 mature mango trees (*Mangifera indica* L., cv. ‘Ewais’) grafted onto Sukari rootstock. The trees, all five years old, were cultivated in a private mango orchard located in the Wadi Natrun region of El-Behera Governorate, Egypt (30.375883°N, 30.346066°E). The trees were planted at a spacing of 5 × 5 m. The surface soil layer (0–15 cm) was classified as loamy sand, with a particle distribution of 58% medium sand, 12% fine sand, and 5.7% clay. The soil reaction was mildly alkaline, with a pH value of 7.98. Electrical conductivity measured in the saturated paste extract was 1.23 dS m^−1^, reflecting a moderate salinity status. The sodium adsorption ratio (SAR) was 3.65, indicating a slight sodicity hazard, though not at a level that would severely affect soil structure or plant growth (Supplementary Table S1).

All trees were irrigated with naturally saline water, and the orchard at Wadi El-Natrun was managed under a controlled irrigation system throughout both growing seasons. Meteorological data, including average monthly temperature ranges and rainfall for both seasons, are summarized in Supplementary Table S2. In each year of the study, irrigation water was sampled 12 times, once each month, and the annual average salinity was calculated using portable electrical conductivity (EC) meter. The mean salinity was 1,350 mg L⁻¹ in the first season and 1,500 mg L⁻¹ in the second season. The EC meter was calibrated prior to each use using standard calibration solutions, in accordance with the manufacturer’s instructions. To ensure measurement precision, recalibration was performed regularly throughout the analysis period. The treatments included polyethylene glycol (PEG6000) applied at concentrations of 15 mM (T_1_) and 30 mM (T_2_), and proline applied at 8 mM (T_3_) and 13 mM (T_4_). These were administered both individually and in combination as follows: T_1_ with T_3_ (T_5_), T_2_ with T_4_ (T_6_), T_1_ with T_4_ (T_7_), and T_2_ with T_3_ (T_8_). A ninth treatment (T_9_) served as the control, involving trees subjected to the same salinity conditions without the application of either PEG or proline. The treatment structure was as follows:

T_1_: PEG 15 mM 

T_2_: PEG 30 mM

T_3_: Proline 8 mM

T_4_: Proline 13 mM

T_5_: PEG 15 mM + Proline 8 mM

T_6_: PEG 30 mM + Proline 13 mM

 T_7_: PEG 15 mM + Proline 13 mM

T_8_: PEG 30 mM + Proline 8 mM

T_9_: Control

All treatments were applied to trees receiving regular irrigation with a nearly constant salinity level, as previously described. In each growing season, treatments were replicated three times, with each replicate consisting of two mango trees. This resulted in six trees per treatment and a total of 54 experimental trees (9 treatments × 3 replicates × 2 trees). PEG and proline treatments were applied twice per season, during the pre-flowering (within the first week of February) and the fruit set stage (within the last week of May). PEG was applied as a soil drench at a rate of 3 L per tree, while proline was foliar sprayed using a handheld mist sprayer in early morning. The spray was continued until full leaf surface coverage was achieved, approaching the point of runoff, with an average application volume of approximately 10 L per tree.

### Measurements

Fifteen physiological, biochemical, hormonal, and agronomic traits were measured across two growing seasons. For each treatment, six trees of similar shape and size were selected and tagged; three of them were used for sample collection for physiological and biochemical analyses, while the remaining three were used for field-based morphological observation. Each tree was divided into four directional quadrants (east, north, west and south), 20 one-year-old shoots were randomly selected and tagged at each tree direction in October of both study seasons. All selected shoots were approximately similar in length, as they emerged during the same growth cycle. All physiological and biochemical assays were conducted on leaves harvested in the last week of July, coinciding with peak vegetative activity. All analyses were conducted using three independent biological replicates to ensure the reliability and reproducibility of the results. Fully expanded leaves (third to fifth position from the shoot apex) were collected to perform the following analysis:

## Physiological parameters

**Relative water content (RWC) **The relative water content (RWC) was assessed using a modified protocol based on the method described by Smart and Bingham [31]. Fully expanded leaves were sampled from each treatment group, and their fresh weight (FW) was recorded promptly following collection to ensure measurement accuracy. The samples were then submerged in distilled water and kept in the dark at room temperature for 8 h to reach full turgidity, after which they were gently blotted dry and weighed again to obtain the turgid weight (TW). Subsequently, the leaves were dried in a hot-air oven at 70 °C for 48 h to a constant weight to determine their dry weight (DW). RWC was calculated using the formula: RWC (%) = [(FW − DW)/(TW − DW)] × 100.

**Membrane Stability Index (MSI)** Membrane Stability Index (MSI) was determined using the conductivity method after incubating leaf discs in distilled water at two different temperatures to assess electrolyte leakage according to Demidchik et al. [32]. To accomplish this, leaf samples were rinsed with deionized water to remove surface contaminants and then cut into uniform discs, which were subsequently divided into two equal portions. One set was incubated in distilled water at 25 °C, while the other was exposed to a water bath at 45 °C for a fixed duration. Electrical conductivity of the bathing solutions was measured using a conductivity meter after each treatment, with the first reading (C_1_) recorded following the 25 °C treatment and the second (C_2_) after boiling the same samples at 100 °C for 10 min to ensure complete membrane disruption. MSI was calculated using the formula: MSI (%) = [1 - (C_1_/C_2_)] × 100.

### Enzymatic antioxidant activity

A total of 2 g of mango leaf tissue were homogenized in 10 mL of 0.1 M phosphate buffer adjusted to pH 6.8. The homogenate was centrifuged at 20,000 rpm for 20 min at 2 °C using a refrigerated centrifuge. The resulting clear supernatant, containing soluble enzyme fractions, was collected and used as the enzymatic extract for subsequent analysis, following the method described by Mukherjee et al. [33].

### Peroxidase (POD) activity

Peroxidase (POD) activity was assayed by monitoring guaiacol oxidation in the presence of hydrogen peroxide (H₂O₂) [34]. The reaction mixture contained phosphate buffer (pH 6.5), guaiacol (as a hydrogen donor), H₂O₂ (as a substrate), and enzyme extract. Upon oxidation, guaiacol is converted to tetraguaiacol, a brown product with a maximum absorption of 470 nm. The increase in absorbance at this wavelength is recorded over time to determine the initial reaction rate. POD activity is calculated using the extinction coefficient of tetraguaiacol, where a unit (U) of enzyme is defined as the amount that catalyses the formation of 1 µmol of tetraguaiacol per minute. Results were expressed as U mg⁻¹.

### Polyphenol oxidase (PPO) activity

Polyphenol oxidase (PPO) activity was measured using catechol as substrate, with absorbance recorded at 420 nm [35]. This protocol involves preparing a reaction mixture containing phosphate buffer (pH 6.5) and catechol as a substrate. The reaction is initiated by adding the enzyme extract, with o-quinone formation monitored by measuring the increase in absorbance at 420 nm for one minute. PPO activity is calculated based on the initial linear rate of absorbance change. The enzyme activity unit (U) is defined as the amount of PPO required to produce 1 µmol of o-quinone per minute. Results were expressed as U mg⁻¹.

### Non-enzymatic antioxidants

Ascorbate concentration was quantified via 2,6-dichlorophenolindophenol (DCPIP) titration [36] measuring the reduction of DCPIP at 520 nm. Fresh leaf tissue (1 g) was homogenized in 10 mL ice-cold 3% metaphosphoric acid, centrifuged (10,000 rpm, 15 min, 4 °C), and the supernatant collected. For titration, 5 mL supernatant was mixed with 2 mL acetate buffer (pH 3.8) and titrated with standardized 0.1 mM DCPIP solution to a persistent faint pink endpoint (15 s). A blank (metaphosphoric acid + buffer) was run in parallel. All procedures were performed under dim light to prevent oxidation. Results were expressed as mg g⁻¹ FW.

### Photosynthetic pigments

Total chlorophyll (a + b) was extracted from fresh leaf tissue (0.25 g) using 20 mL acetone (80%) [37]. The extract absorbance was recorded at 646 and 663 nm using a double-beam spectrophotometer (JENWAY 6300). Chlorophyll concentrations (mg g^−1^ FW) were calculated using the established equations:

Chlorophyll a = (12.21 × A₆₆₃) − (2.81 × A₆₄₆).

Chlorophyll b = (20.13 × A₆₄₆) − (5.03 × A₆₆₃).

Total chlorophyll = Chlorophyll a + Chlorophyll b.

## Osmolytes and metabolic indicators

### Total soluble sugars (TSS)

Total soluble sugars (TSS) were chemically determined using the anthrone-sulfuric acid method [38] with modifications. Leaf tissue (100 mg) was boiled in 5 mL of 80% ethanol at 80 °C for 30 min. After cooling, the extract was filtered through Whatman No. 1 filter paper, and the residue was re-extracted twice with 5 mL of 80% ethanol. Combined filtrates were evaporated to dryness and redissolved in 5 mL distilled water. For quantification, 0.5 mL extract was mixed with 3 mL ice-cold anthrone reagent (0.2% in concentrated H₂SO₄), heated at 100 °C for 10 min, and cooled. Absorbance was measured at 625 nm using a spectrophotometer and calibrated against glucose standards (0–100 µg mL^−1^). Results were expressed as mg glucose equivalents per g dry weight (mg g⁻¹ DW).

### Proline concentration

Proline accumulation was quantified using an optimized acid-ninhydrin assay adapted from Bates et al. [39] with some modifications. Fresh mango leaf tissue (0.5 g) was homogenized in 3% sulfosalicylic acid and centrifuged at 10,000 rpm for 10 min at 4 °C. The supernatant was combined with acid-ninhydrin reagent (1.25 g ninhydrin in 30 mL glacial acetic acid and 20 mL 6 M phosphoric acid) and glacial acetic acid in equal volumes (1:1:1 ratio). After 60 min incubation at 95 °C in a water bath, the reaction was terminated on ice, and the chromophore was extracted with toluene. Absorbance of the toluene phase was measured at 520 nm using a UV-Vis spectrophotometer, with proline concentrations calculated against a L-proline standard curve (0-100 µM). Results were expressed as mg g⁻¹ FW.

### Hormonal analysis

The auxin-abscisic acid ratio (IAA/ABA) was determined using high-performance liquid chromatography (HPLC) following established protocols [40] with some modifications according to Zhang et al. [41]. Fresh leaf tissue (200 mg) was extracted in 80% methanol containing 1% acetic acid and 0.5 mM butylated hydroxytoluene as an antioxidant, followed by centrifugation at 12,000 rpm for 15 min at 4 °C. The supernatant was concentrated under nitrogen gas and resuspended in HPLC-grade methanol. Separation was achieved using a C18 reverse-phase column (4.6 × 250 mm, 5 μm) with a mobile phase of 0.1% formic acid in water (A) and acetonitrile (B) at 0.8 mL min flow rate. IAA and ABA were detected at 280 nm and 260 nm, respectively using a diode array detector. Quantification was performed against authentic standards prepared from pure IAA and ABA solutions (0.1–100 µg mL^−1^). The method closely followed the protocol described in Zhang et al. [41], including column conditioning, solvent purity checks, and retention time verification for peak identification. Internal standards were used where applicable to correct for recovery efficiency.

### Vegetative and reproductive growth parameters

Vegetative and reproductive traits were evaluated to assess salinity stress responses in mango trees. Shoot elongation (cm) was measured on tagged branches using a calibrated measuring tape, while the number of flushes per branch was recorded to monitor regrowth frequency. Leaf area (cm²) was calculated using the length × width × correction factor according to Haseeb et al. [42]. Fruit set percentage was calculated as the ratio of retained fruits to total flowers per panicle at harvest [43], reflecting fertilization success under stress. At commercial maturity (August 17 and 22 in consecutive seasons), individual fruit weight (g) was measured using a precision balance, and total yield per tree (kg) was derived by multiplying fruit count by average fruit weight [44].

### Experimental design and statistical analysis

The experiment was arranged in a randomized complete block design (RCBD) with nine treatments and six replications per treatment, with data from each growing season analysed independently using one-way ANOVA in R (version 4.4.2) [45] at a 95% confidence level. The analysis accounted for block effects while focusing on treatment comparisons, with significant differences (*p* < 0.05) further analysed using Duncan’s multiple range test [46] for all pairwise comparisons among the nine treatments. Model assumptions were validated using the gvlma test (Global Validation of Linear Model Assumptions) to comprehensively assess linear model requirements including skewness, kurtosis, and heteroscedasticity. Non-normal data were transformed using square root or logarithmic transformations when necessary. Principal component analysis (PCA) was conducted using the SRplot tool [47]. Heatmap visualization was performed using the Clustvis tool [48]. Pearson correlation analysis was performed using also the R statistical software, version 4.4.2 [45], employing appropriate packages for correlation matrix computation and visualization.

## Results

### Physiological parameters

**Fig. 1:**
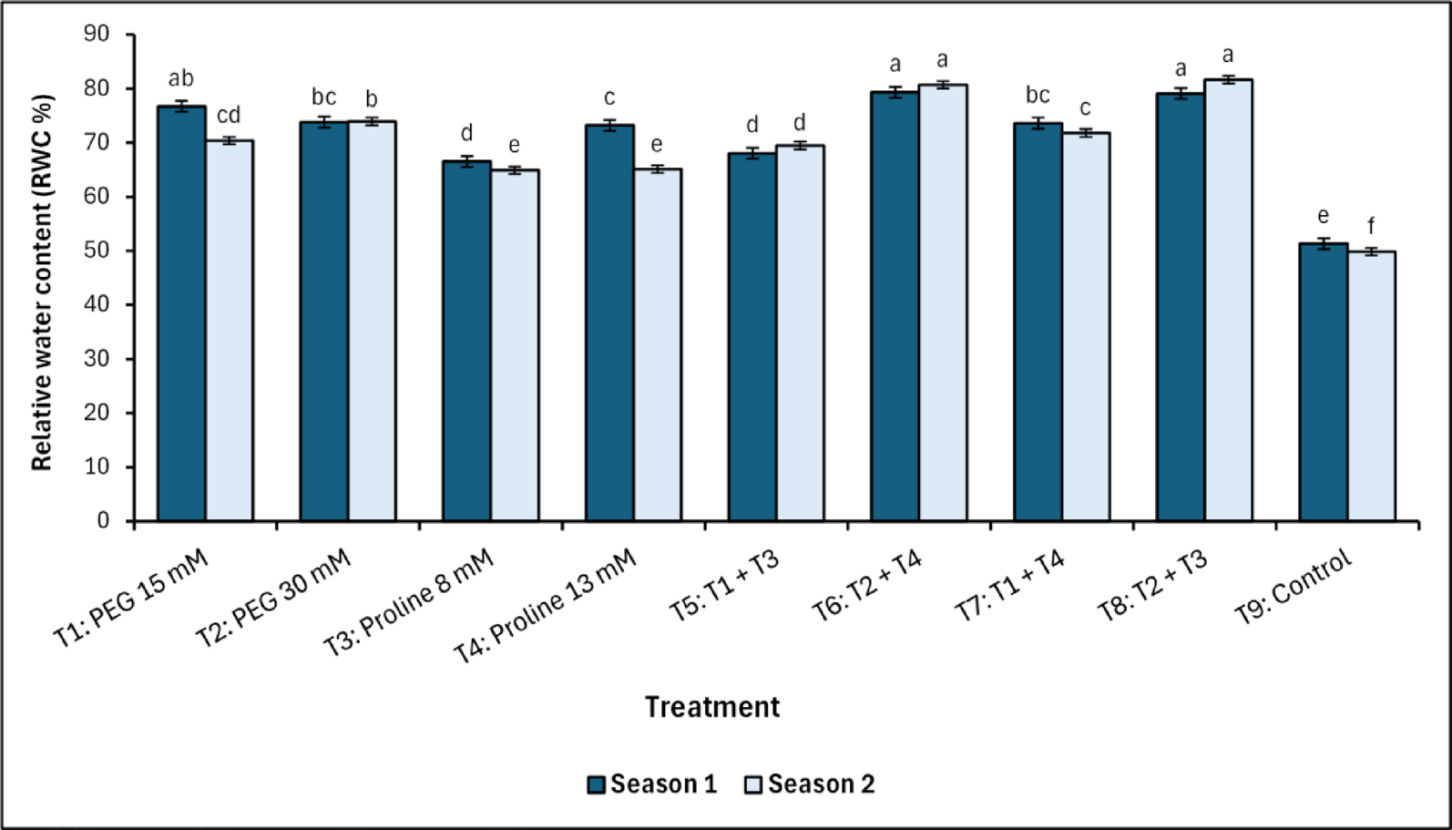
Changes in relative water content (RWC) in response to treatments with polyethylene glycol (PEG), proline, and their combinations, alongside the control. Data from each season were analysed separately using Duncan’s multiple range test (*p* < 0.05) identifying statistically similar means (shared lowercase letters within a season). Vertical bars reflect the standard error (± SE), based on three biological replicates (*n* = 3)

A significant increase in relative water content (RWC) was observed in treatments T_6_ (PEG 30 mM + Proline 13 mM) and T_8_ (PEG 30 mM + Proline 8 mM), as illustrated in Fig. 1. The data reveals a clear variation in RWC across treatments, with T_6_ (79.31^a^ % in Season 1 and 80.71^a^ % in Season 2) and T_8_ (79.11^a^ % in Season 1 and 81.66^a^ % in Season 2) forming distinct high-performance groups, consistently maintaining RWC values nearly 30% points above the control treatment (T_9_; 51.34^e^ and 49.88^f^ % in the first and second season, respectively).

**Fig. 2:**
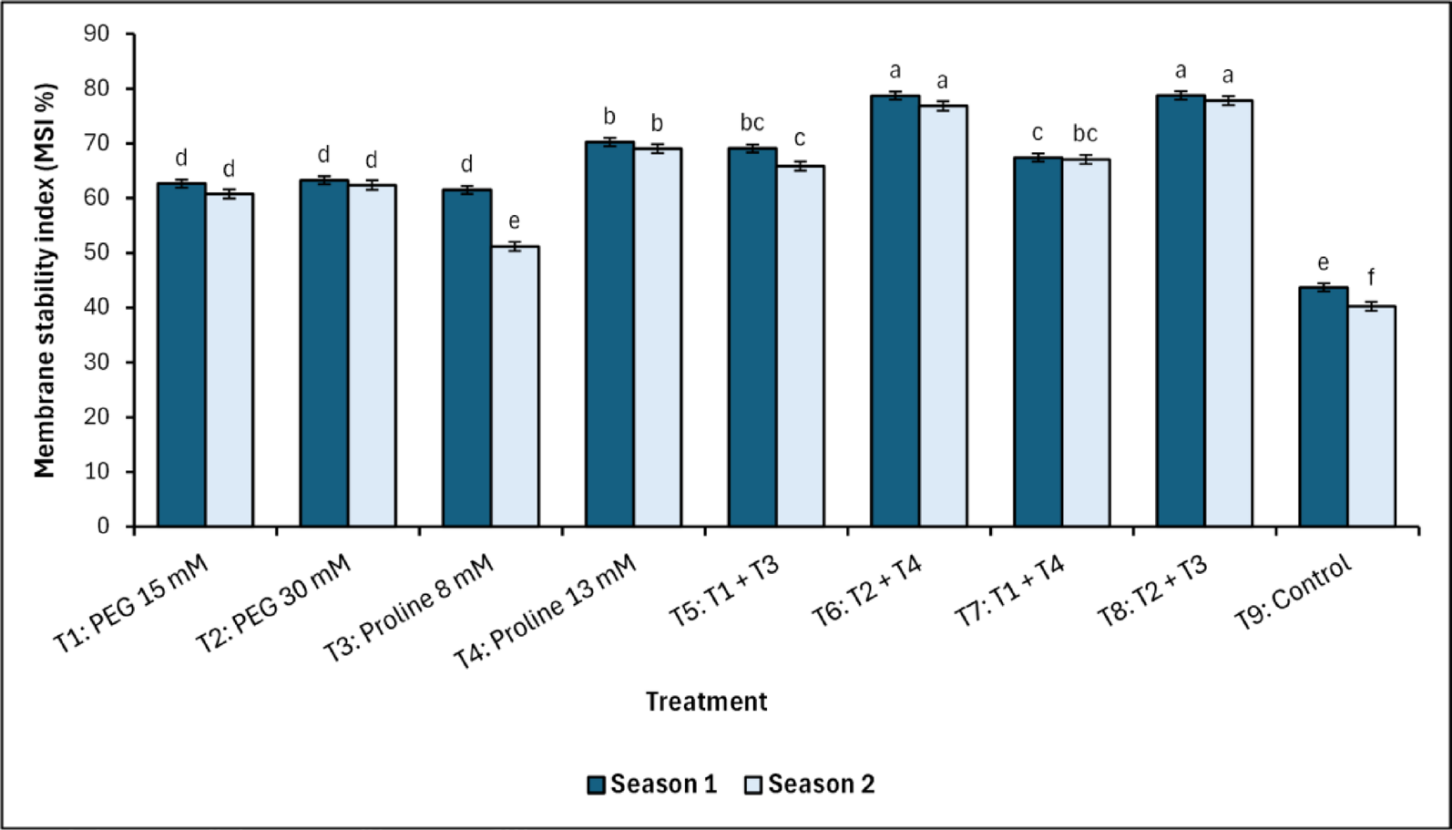
Changes in membrane stability index (MSI) in response to treatments with polyethylene glycol (PEG), proline, and their combinations, alongside the control. Data from each season were analysed separately using Duncan’s multiple range test (*p *< 0.05) identifying statistically similar means (shared lowercase letters within a season). Vertical bars reflect the standard error (± SE), based on three biological replicates (*n* = 3)

All treatments, without exception, had a positive effect on increasing the relative water content of leaves under salt stress compared to control in both seasons of the study. Considering the closest values to the control treatment (51.34^e^ % in the 1st season), we found that the 8 mM proline (T_3_) treatment recorded the closest values of RWC in the first season (66.51^d^ %), and these results were confirmed by the second season values (49.88^f^ % for the control and 64.90^e^ % for the T_3_ treatment).

Treated and untreated (control) mango trees followed the same behaviour when estimating membrane stability index (MSI), which showed that the best treatment was T_8_ (combined treatment with polyethylene glycol and proline), which recorded values equal to 78.78^a^ and 77.84^a^ % in the first and second season, respectively (Fig. 2). In contrast, the control treatment had the lowest significant (*p* < 0.05) cell membrane stability in both seasons of the study (43.71^e^ and 40.23^f^ % in the first and second season, respectively).

### Enzymatic and non-enzymatic antioxidant activity

**Fig. 3:**
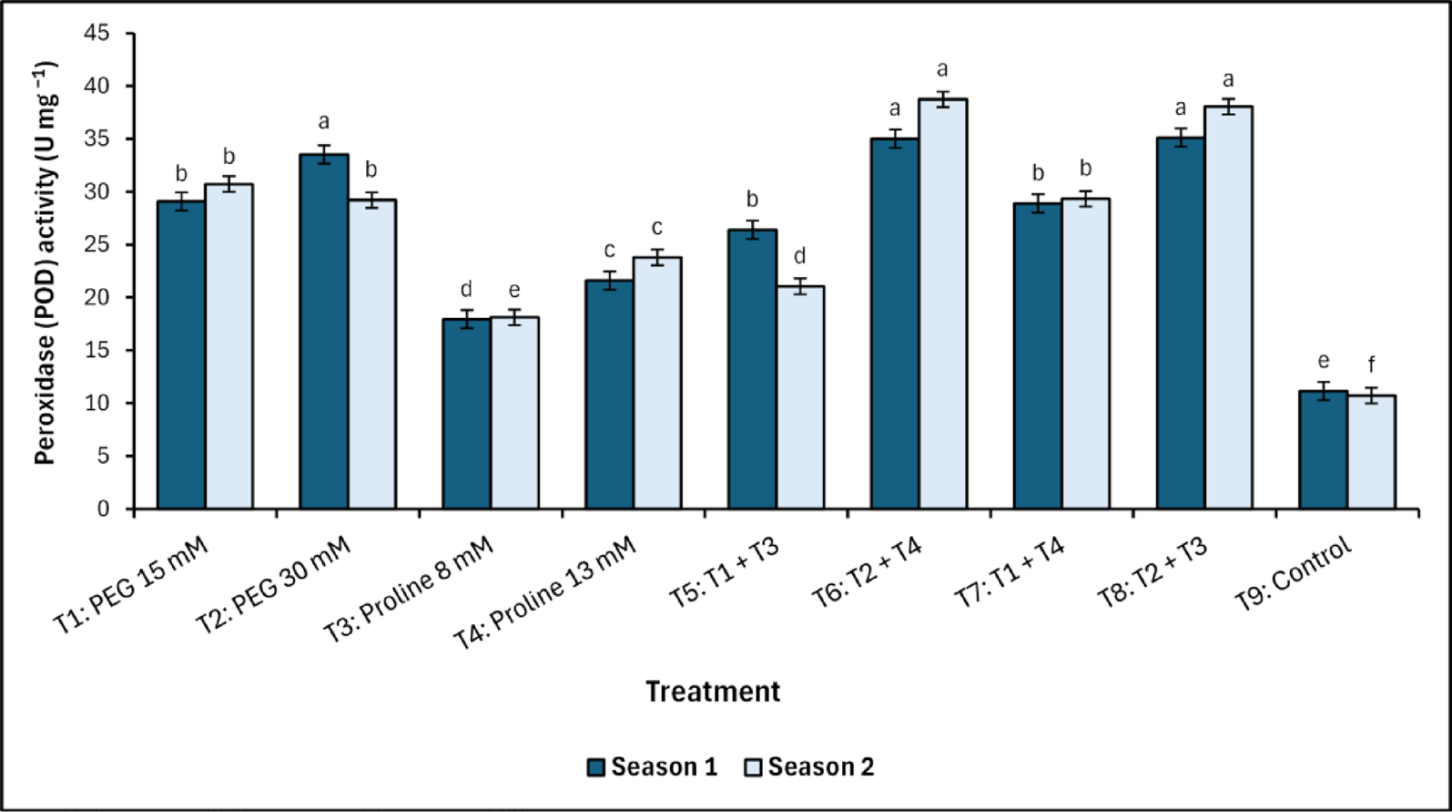
Changes in peroxidase (POD) activity in response to treatments with polyethylene glycol (PEG), proline, and their combinations, alongside the control. Data from each season were analysed separately using Duncan’s multiple range test (*p* < 0.05) identifying statistically similar means (shared lowercase letters within a season). Vertical bars reflect the standard error (± SE), based on three biological replicates (*n* = 3)

In general, POD enzyme activity increased with spraying with PEG and proline in combination, especially T_6_ and T_8_ (Fig. 3).

Treatment T_6_ recorded values equal to 35.01^a^ U mg ^−1^ in the first season and 38.73^a^ U mg ^−1^ in the second season, while treatment T_8_ recorded similar values equal to 35.11^a^ and 38.05^a^ U mg ^−1^ in the first and second season, respectively. On the other hand, untreated trees (control) recorded the lowest significant (*p* < 0.05) values of 11.14^e^ and 10.71^f^ U mg^−1^ for the first and second season, respectively.

**Fig. 4:**
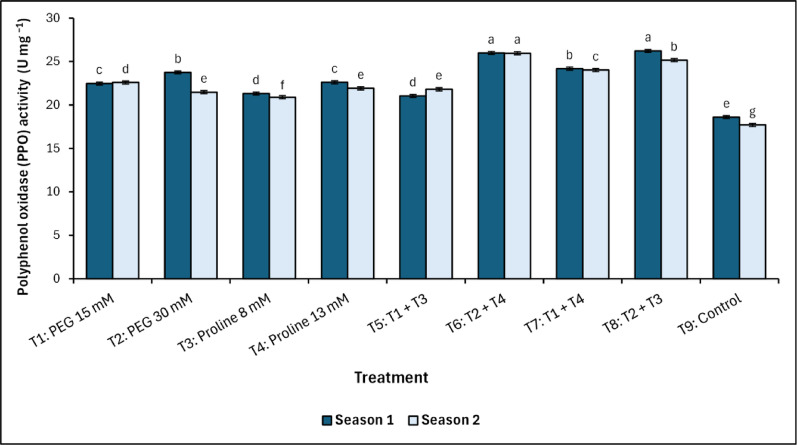
Changes in polyphenol oxidase (PPO) activity in response to treatments with polyethylene glycol (PEG), proline, and their combinations, alongside the control. Data from each season were analysed separately using Duncan’s multiple range test (*p* < 0.05) identifying statistically similar means (shared lowercase letters within a season). Vertical bars reflect the standard error (± SE), based on three biological replicates (*n* = 3)

The analysis of polyphenol oxidase (PPO) activity under salinity stress revealed significant variations among the different treatments in both growing seasons (Fig. 4). The control group (T_9_) exhibited the lowest significant (*p* < 0.05) PPO activity, recording 18.6^e^ U mg ^−1^ and 17.7^g^ U mg ^−1^ in the first and second seasons, respectively. In contrast, T_8_ (PEG + proline combination) demonstrated the highest significant (*p* < 0.05) PPO activity, with values of 26.22^a^ U mg ^−1^ and 25.16^b^ U mg ^−1^ across the two seasons.

**Fig. 5:**
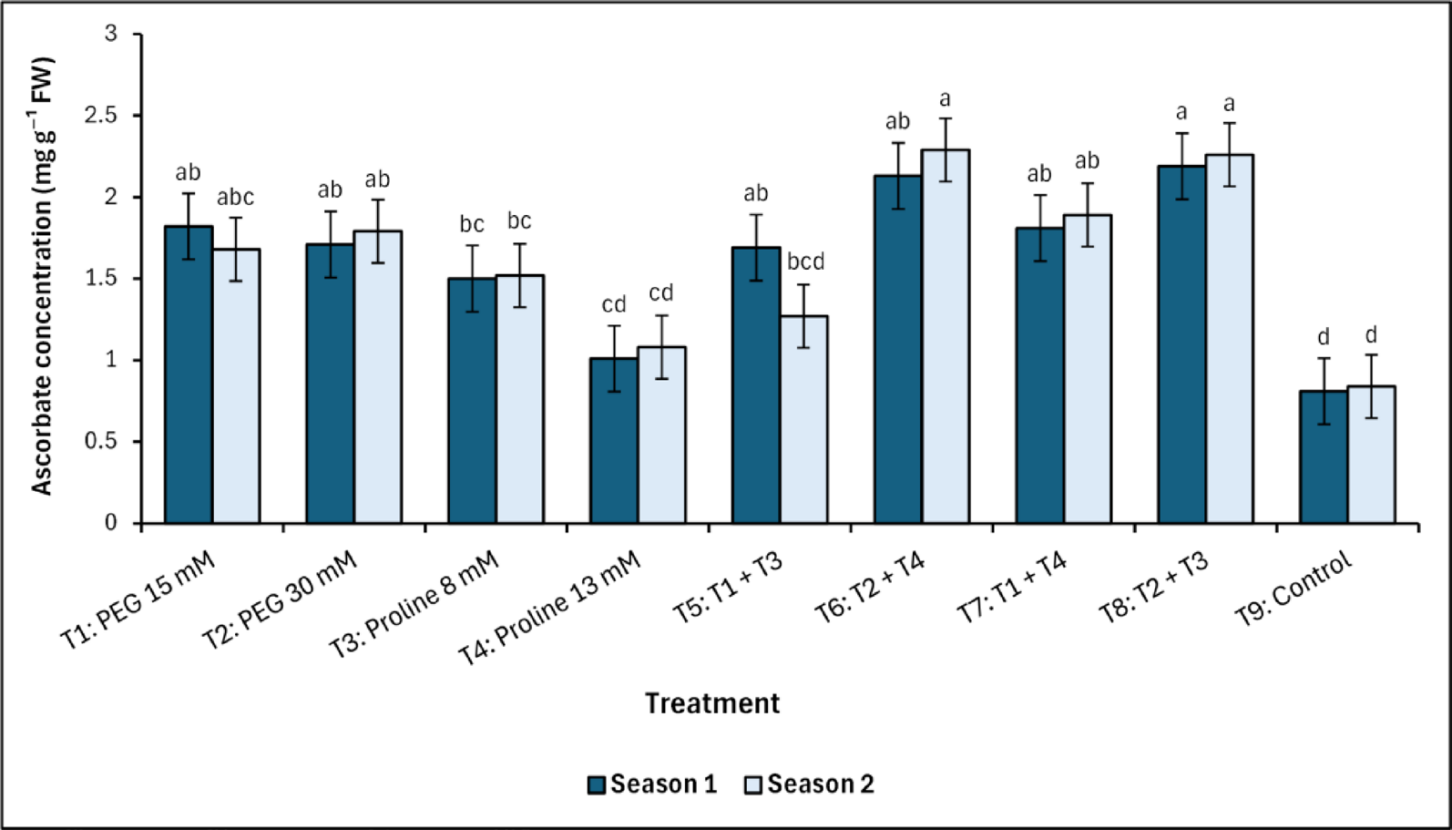
Changes in ascorbate concentration in response to treatments with polyethylene glycol (PEG), proline, and their combinations, alongside the control. Data from each season were analysed separately using Duncan’s multiple range test (*p* < 0.05) identifying statistically similar means (shared lowercase letters within a season). Vertical bars reflect the standard error (± SE), based on three biological replicates (*n* = 3)

This was closely followed by T_6_, which maintained consistently high PPO levels at 25.98^a^ U mg ^−1^ (first season) and 25.95^a^ U mg ^−1^ (second season). In the same pattern, the concentration of the non-enzymatic ascorbate increased with both T_6_ and T_8_ treatments, which included the combined spraying with PEG and proline (Fig. 5). The T_6_ (PEG 30 mM + Proline 13 mM) treatment recorded values of 2.13^ab^ mg g^−1^ fresh weight in the first season and 2.29^a^ mg g^−1^ fresh weight in the second season, while the T_8_ (PEG 30 mM + Proline 8 mM) treatment recorded values of 2.19^a^ mg g^−1^ fresh weight and 2.26^a^ mg g^−1^ fresh weight in the first and second season, respectively. On the other hand, the control treatment recorded the lowest values of 0.81^d^ and 0.84^d^ mg g^−1^ fresh weight for the first and second season, respectively.

### Photosynthetic pigments

**Fig. 6:**
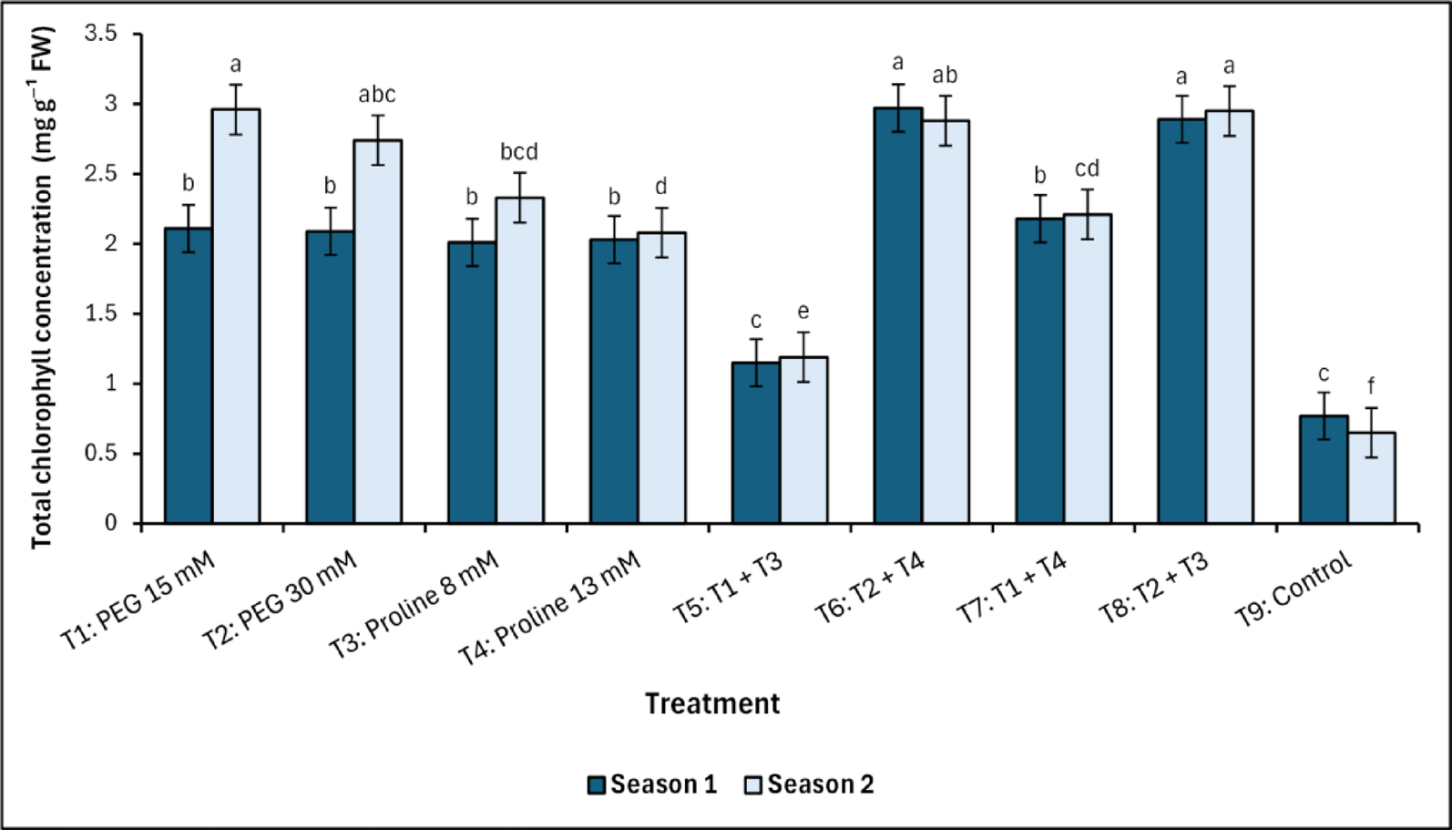
Changes in total chlorophyll in response to treatments with polyethylene glycol (PEG), proline, and their combinations, alongside the control. Data from each season were analysed separately using Duncan’s multiple range test (*p* < 0.05) identifying statistically similar means (shared lowercase letters within a season). Vertical bars reflect the standard error (± SE), based on three biological replicates (*n* = 3)

There were differences between the response of growing mango trees under salt stress conditions (Fig. 6), with the control treatment recording the lowest values of leaf chlorophyll concentration in both seasons of the study (0.77^c^ and 0.65^f^ mg g^−1^ FW in the first and second season, respectively). Regarding the PEG and proline treatments, T_5_ (PEG 15 mM + Proline 8 mM) treatment recorded the lowest concentration of chlorophyll in mango leaves, the closest values to the control treatment, growing under salinity conditions in both seasons with values of 1.15^c^ and 1.19^e^ mg g^−1^ FW, respectively, while T_1_ (PEG 15mM), T_6_ (PEG 30 mM + Proline 13 mM), and T_8_ (PEG 30 mM + Proline 8 mM) treatments recorded the highest concentrations in the second (2.96^a^ mg g^−1^ FW), first (2.97^a^ mg g^−1^ FW), and second (2.95^a^ mg g^−1^ FW) seasons, respectively (Fig. 6).

### Osmolytes and metabolic indicators

**Fig. 7:**
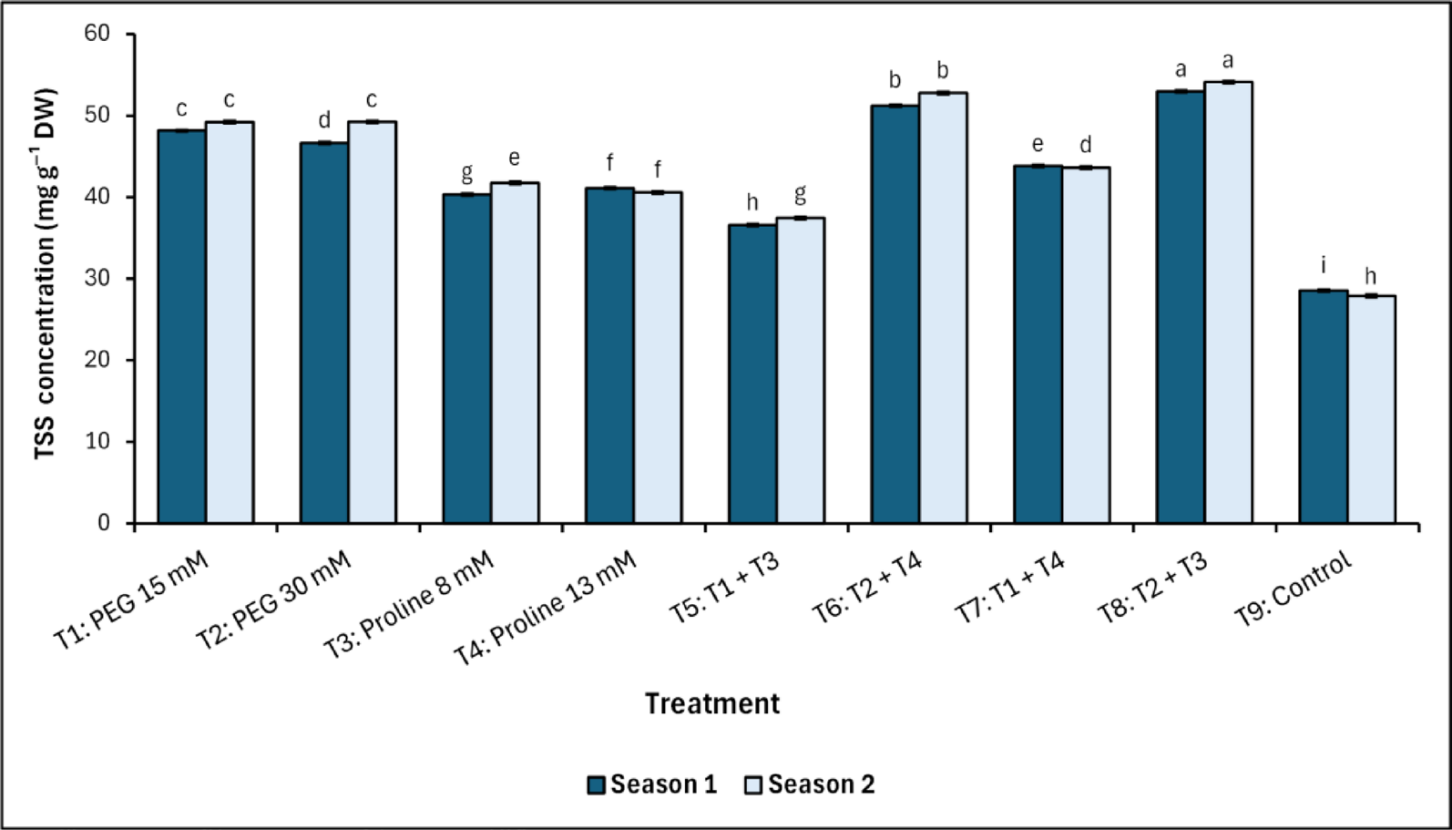
Changes in total soluble sugars (TSS) in response to treatments with polyethylene glycol (PEG), proline, and their combinations, alongside the control. Data from each season were analysed separately using Duncan’s multiple range test (*p* < 0.05) identifying statistically similar means (shared lowercase letters within a season). Vertical bars reflect the standard error (± SE), based on three biological replicates (*n* = 3)

The data presented in Fig. 7 shows that there are differences in the concentration of total soluble sugars (TSS) in mango leaves exposed to salt stress as a result of treatment with PEG and proline. The results showed that PEG treatment combined with proline recorded the most significant (*p* < 0.05) concentration of TSS in mango leaves (T_8_) in the two seasons of the study, recording values of 52.98^a^ and 54.11^a^ mg g^−1^ FW in the first and second season, respectively. While the control recorded the lowest significant values in both seasons (28.55^i^ and 27.91^h^ mg g^−1^ FW in the first and second season, respectively).

**Fig. 8:**
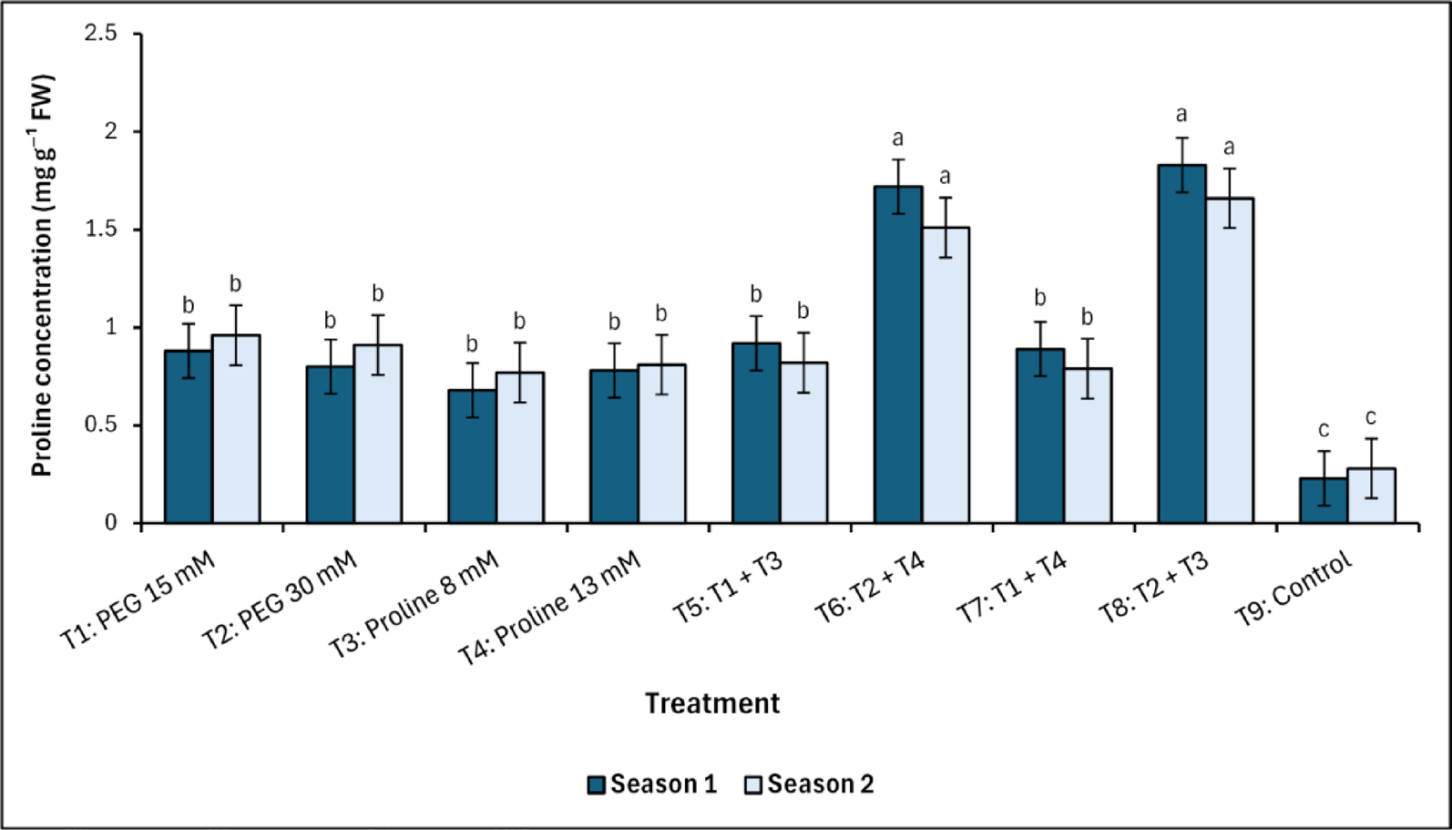
Changes in proline concentration in response to treatments with polyethylene glycol (PEG), proline, and their combinations, alongside the control. Data from each season were analysed separately using Duncan’s multiple range test (*p* < 0.05) identifying statistically similar means (shared lowercase letters within a season). Vertical bars reflect the standard error (± SE), based on three biological replicates (*n* = 3)

The internal proline concentration in mango leaves varied significantly under salinity stress across both seasons of this study, with the significantly (*p* < 0.05) highest accumulation observed in T_6_ (PEG 30 mM + Proline 13 mM) and T_8_ (PEG 30 mM + Proline 8 mM), without observing any significant differences between them (Fig. 8). Also, the data indicated that there were no significant differences between the first fifth treatments (T_1_ to T_5_). On the other hand, T_9_ (control) recorded the lowest significant (*p* < 0.05) values in the first and second season (0.23^c^ and 0.28^c^ mg g^−1^ FW, respectively).

### Hormonal analysis

**Fig. 9:**
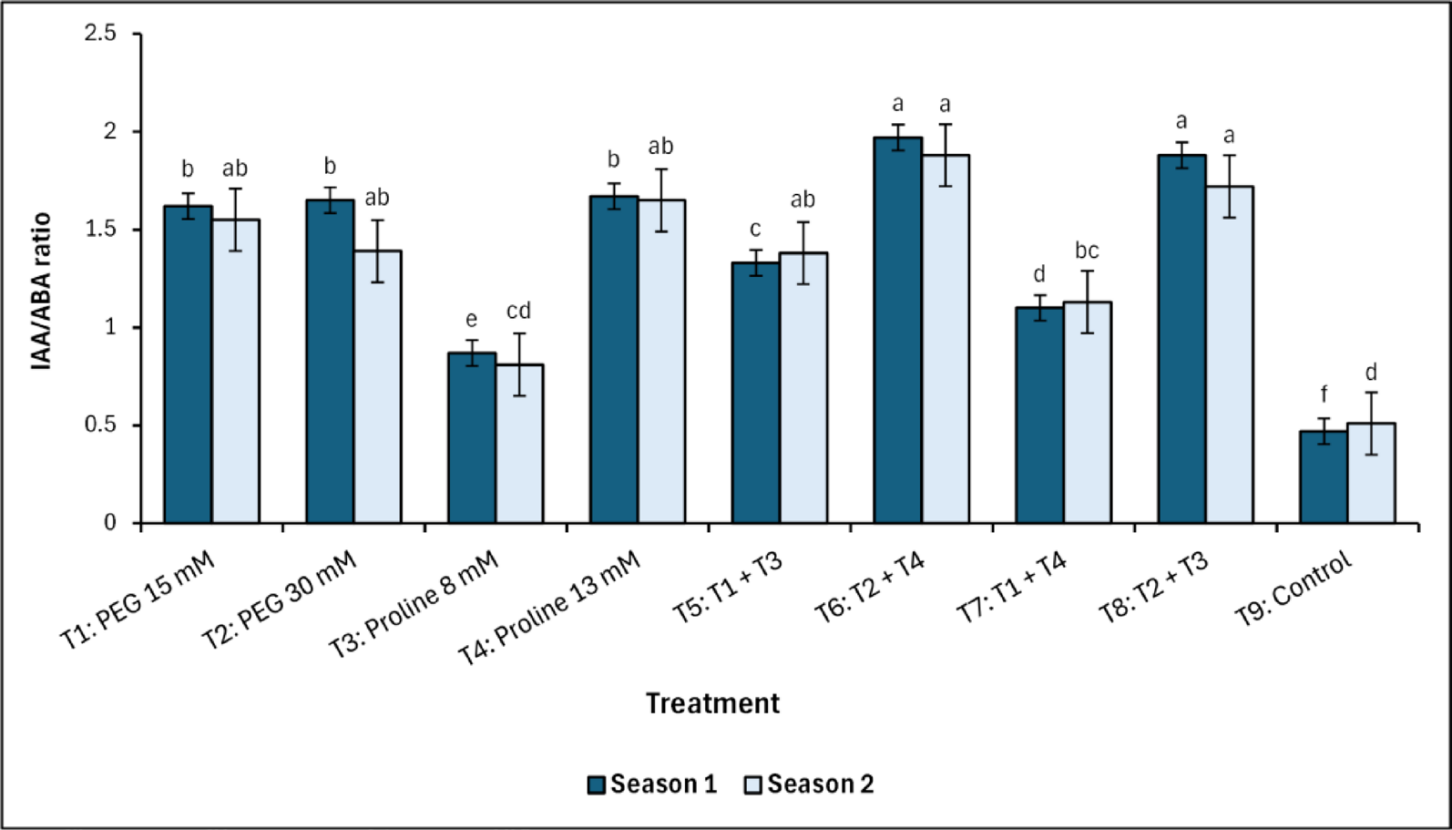
Changes in IAA/ABA ratio in response to treatments with polyethylene glycol (PEG), proline, and their combinations, alongside the control. Data from each season were analysed separately using Duncan’s multiple range test (*p* < 0.05) identifying statistically similar means (shared lowercase letters within a season). Vertical bars reflect the standard error (± SE), based on three biological replicates (*n* = 3)

Across both growing seasons, hormonal analysis revealed a significant increase in IAA/ABA ratios in all treated groups compared to the control (Fig. 9 and Supplementary Figure S1). Notably, T_6_ (PEG 30 mM + Proline 13 mM) recorded the highest ratios, with values of 1.97^a^ and 1.88^a^ in the first and second seasons, respectively. Similarly, T_8_ (PEG 30 mM + Proline 8 mM) exhibited elevated ratios of 1.88^a^ in the first season and 1.72^a^ in the second season. In contrast, the control treatment exhibited the lowest statistically significant (*p* < 0.05) ratio, measuring 0.47^f^ in the first season and 0.51^d^ in the second season.

### Vegetative and reproductive growth parameters

Vegetative and reproductive growth parameters were also monitored, including shoot length (cm), flushes number per branch, leaf area (cm²), fruit set percentage, fruit weight (g), and yield per tree (kg). The data presented in Table 1 indicates that the greatest significant (*p* < 0.05) increase in shoot length was observed in treatments T_6_ and T_8_, with recorded values of 73.22^a^ and 72.85^a^ cm, respectively in the 1^st^ season. In contrast, the lowest statistically significant (*p* < 0.05) value (52.44^e^ cm) was associated with the control treatment (T_9_), a trend that was consistently observed in the second season as well.

Regarding the number of flushes, treatment T_8_ (PEG 30 mM + Proline 8 mM) also recorded the highest significant (*p* < 0.05) value (3.70^a^) compared to the control (T_9_), which exhibited the lowest significant (*p* < 0.05) value (1.61^d^) in the first season. This result was confirmed in the second season. Similarly, treatment T_8_ produced the largest significant (*p* < 0.05) leaf area (139.07^a^ cm²), whereas the control treatment (T_9_) recorded the lowest significant (*p* < 0.05) value (79.34^i^ cm²) in the first season, a trend that was observed in the second season as well with values equal to 137.60^a^ cm² with T_8_ and 81.28^h^ cm² with T_9_ (control).

**Tale 1b:** Changes in shoot length, flushes number, and leaf area in response to treatments with polyethylene glycol (PEG), proline, and their combinations, alongside the control

Treatment	Shoot length (cm)	Flushes No.	Leaf area (cm²)
	1st season		
T_1_: PEG 15 mM	69.94 b	3.01 ab	128.19 d
T_2_: PEG 30 mM	70.13 b	3.07 ab	128.78 c
T_3_: Proline 8 mM	67.69 c	2.71 bc	101.17 g
T_4_: Proline 13 mM	65.18 d	2.02 cd	96.66 h
T_5_: T_1_ + T_3_	67.19 c	2.63 bc	104.29 f
T_6_: T_2_ + T_4_	73.22 a	3.44 ab	131.46 b
T_7_: T_1_ + T_4_	69.71 b	2.71 bc	124.82 e
T_8_: T_2_ + T_3_	72.85 a	3.70 a	139.07 a
T_9_: Control	52.44 e	1.61 d	79.34 i
	2nd season		
T_1_: PEG 15 mM	69.80 b	3.15 ab	122.45 d
T_2_: PEG 30 mM	69.93 b	3.21 ab	125.92 c
T_3_: Proline 8 mM	66.93 e	2.81 b	118.12 e
T_4_: Proline 13 mM	64.04 f	2.11 c	95.80 g
T_5_: T_1_ + T_3_	68.15 d	2.55 bc	103.62 f
T_6_: T_2_ + T_4_	72.21 a	3.80 a	137.03 b
T_7_: T_1_ + T_4_	69.13 c	2.57 bc	117.79 e
T_8_: T_2_ + T_3_	72.71 a	3.72 a	137.60 a
T_9_: Control	53.81 g	1.38 d	81.28 h

Data from each season were analysed separately using Duncan’s multiple range test (*p* < 0.05) identifying statistically similar means (shared lowercase letters within a season

From the results of Table 2, the percentage of fruit set peaked significantly (*p* < 0.05) at T_8_ (PE 30 mM + proline 8 mM) with values equal to 59.01^a^ and 61.10^a^ % in the first and second season, respectively, while the lowest significant (*p* < 0.05) values (18.76^i^ and 21.34^i^ %) were recorded with the control treatment in the first and second season, respectively. It is also observed that the highest significant (*p* < 0.05) fruit weight was achieved by control treatment (T_9_) in both seasons of the study, with recorded weights of 365.21^a^ and 381.06^a^ g in the first and second seasons, respectively.

**Table 2:** Changes in fruit set %, fruit weight, and yield in response to treatments with polyethylene glycol (PEG), proline, and their combinations, alongside the control

**Treatment**	**Fruit set %**	**Fruit weight (g)**	**Yield (kg per tree)**
	1st season		
T_1_: PEG 15 mM	48.14 c	318.71 b	33.89 c
T_2_: PEG 30 mM	46.77 d	320.39 b	31.90 d
T_3_: Proline 8 mM	33.84 h	305.67 cd	32.03 d
T_4_: Proline 13 mM	35.11 g	312.11 bc	27.25 g
T_5_: T_1_ + T_3_	40.25 e	319.36 b	29.78 f
T_6_: T_2_ + T_4_	57.02 b	301.88 d	41.76 b
T_7_: T_1_ + T_4_	39.39 f	305.49 cd	31.20 e
T_8_: T_2_ + T_3_	59.01 a	307.61 cd	43.13 a
T_9_: Control	18.76 i	365.21 a	11.76 h
	2nd season		
T_1_: PEG 15 mM	47.40 d	304.14 cd	33.22 c
T_2_: PEG 30 mM	48.27 c	324.26 b	30.56 de
T_3_: Proline 8 mM	35.00 g	298.66 d	29.01 f
T_4_: Proline 13 mM	34.02 h	318.92 bc	26.11 g
T_5_: T_1_ + T_3_	41.92 e	315.78 bc	30.87 d
T_6_: T_2_ + T_4_	59.77 b	290.75 d	43.45 a
T_7_: T_1_ + T_4_	40.26 f	298.18 d	30.21 e
T_8_: T_2_ + T_3_	61.10 a	299.90 d	42.07 b
T_9_: Control	21.34 i	381.06 a	10.44 h

Data from each season were analysed separately using Duncan’s multiple range test (*p* < 0.05) identifying statistically similar means (shared lowercase letters within a season)

However, in terms of yield, the highest significant (*p* < 0.05) value (43.13^a^ kg per tree) was recorded for treatment T_8_ (PEG 30 mM + Proline 8 mM) in the first season. In the second season, the highest significant (*p* < 0.05) yield was observed with treatment T_6_ (43.45^a^ kg per tree), followed by T_8_ (42.07^b^ kg per tree). In contrast, the control treatment consistently recorded the lowest significant (*p* < 0.05) values in both seasons of the study.

#### Principal component analysis (PCA), heatmap visualization, and correlation analysis

**Fig. 10:**
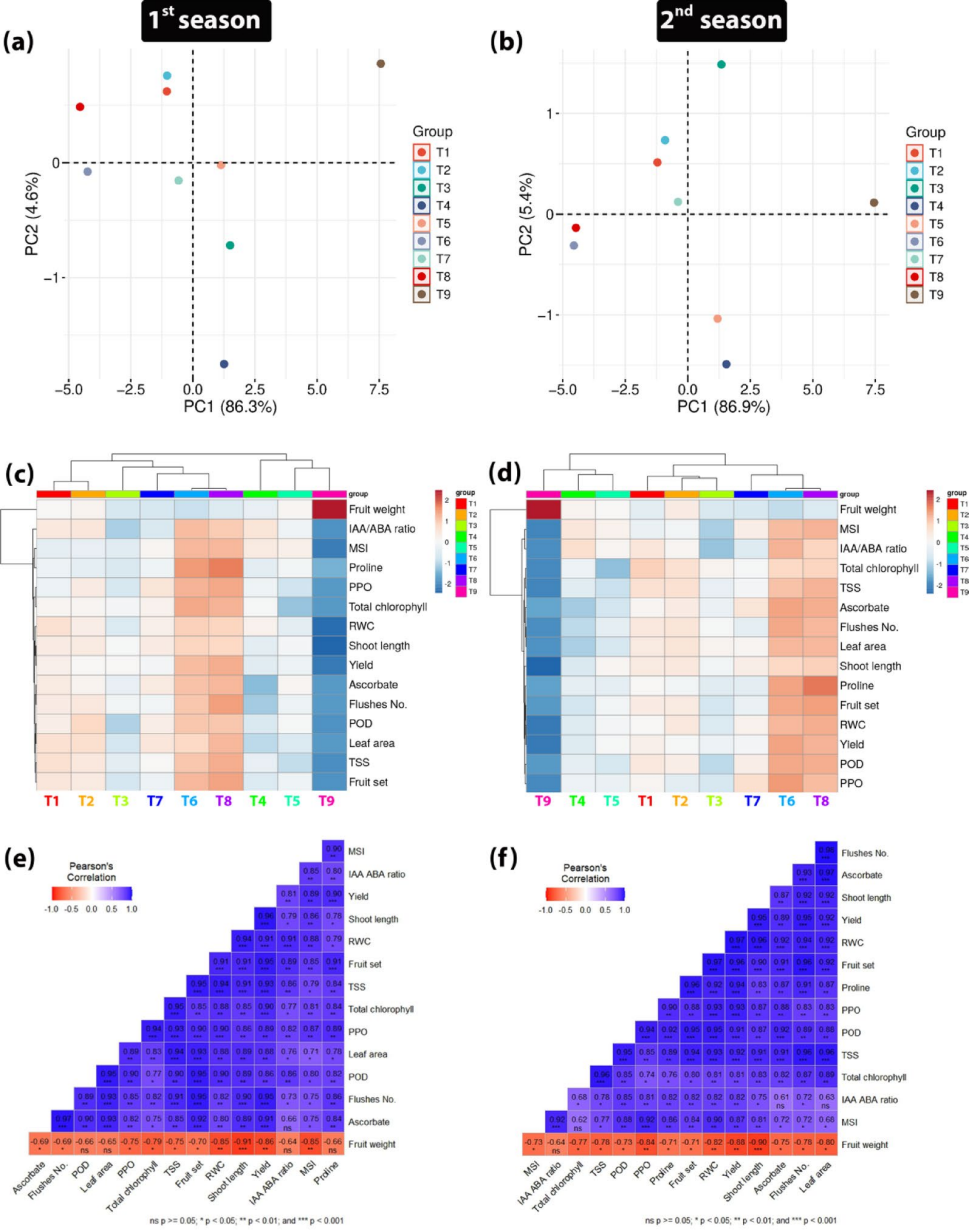
Principal Component Analysis (PCA) scores plot of the nine treatment groups in the first (**a**) and second (**b**) seasons; hierarchical clustering with heatmap visualization of measured parameters across treatments in the first (**c**) and second (**d**) seasons; Pearson’s correlation matrices illustrating relationships among studied parameters in the first (**e**) and second (**f**) seasons

The Principal Component Analysis (PCA) revealed distinct patterns of variance among the nine treatment groups, with notable similarities and subtle differences between the two study seasons. In the first season, the first two principal components (PC1 and PC2) accounted for 86.3% and 4.6% of the total variance, respectively, with a combined explained variance of 90.9% (*p* < 0.001). In the second season, these values shifted slightly to 86.9% for PC1 and 5.4% for PC2, resulting in a combined explained variance of 92.3% (*p* < 0.001). This indicates that PC1 dominated the explained variance in both seasons, capturing most of the dataset’s variability, while PC2 contributed only a minor (though slightly increased) proportion in the second season (Fig. 10a and b). The heat map analysis in both seasons of the study also showed that there was a clear difference and differentiation of the control treatment from the other treatments, and this was clearly visible in the weight of the fruits (Fig. 10c and d). Also, this analysis showed a correlation and similarity between the T_6_ and T_8_ treatments compared to the control in both seasons of the study based on all the studied measurements. Pearson’s correlation analysis revealed a broadly consistent positive interdependence among most measured variables, suggesting synergistic physiological and agronomic relationships within the studied system in both seasons (Fig. 10e and f). However, fruit weight exhibited a distinct negative correlation with all other parameters, a finding that warrants careful interpretation. Notably, this inverse relationship was significant with key traits such as membrane stability index (MSI; *r* = − 0.85, *p <* 0.01 in the 1^st^ season and *r* = − 0.73, *p <* 0.05 in the 2^nd^ season), yield (*r* = − 0.86, *p <* 0.01 in the 1^st^ season and *r* = − 0.88, *p <* 0.01 in the 2^nd^ season), shoot length (*r* = − 0.91, *p <* 0.001 in the 1^st^ season and *r* = − 0.90, *p <* 0.001 in the 2^nd^ season), relative water content (RWC; *r* = − 0.85, *p <* 0.01 in the 1st season and *r* = − 0.82, *p <* 0.01 in the 2^nd^ season), fruit set (*r* = − 0.70, *p <* 0.05 in the 1^st^ season and *r* = − 0.71, *p <* 0.05 in the 2^nd^ season), total soluble solids (TSS; *r* = − 0.75, *p <* 0.05 in the 1^st^ season and *r* = − 0.78, *p <* 0.05 in the 2^nd^ season), total chlorophyll (*r* = − 0.79, *p <* 0.05 in the 1st season and *r* = − 0.77, *p <* 0.05 in the 2^nd^ season), polyphenol oxidase (PPO) activity (*r* = − 0.75, *p <* 0.05 in the 1st season and *r* = − 0.84, *p <* 0.01 in the 2^nd^ season, flushes number (*r* = − 0.69, *p <* 0.05 in the 1st season and *r* = − 0.78, *p <* 0.05 in the 2^nd^ season), and ascorbate (*r* = − 0.69, *p <* 0.05 in the 1^st^ season and *r* = − 0.75, *p <* 0.05 in the 2^nd^ season). The negative correlations between fruit weight and proline, leaf area, and Peroxidase (POD) were significant (*p* < 0.05) only in the second season, with correlation coefficients of − 0.71, − 0.80, and − 0.73, respectively.

In contrast, the negative correlations between fruit weight and the other studied traits, including proline, leaf area, and POD, were not significant (*p* > 0.05) in the first season, with correlation coefficients of *r* = − 0.66, *r* = − 0.65, and *r* = − 0.66, respectively. In addition, the correlation with the IAA to ABA ratio was not significant in either season (*r* = − 0.64, *p* > 0.05).

## Discussion

Mango (*Mangifera indica* L.) is a vital component of Egypt’s horticultural economy. However, like many perennial fruit crops, mango is highly susceptible to salinity stress, which disrupts physiological and biochemical homeostasis by inducing osmotic imbalance, ionic toxicity, and oxidative damage [3, 6]. Understanding and mitigating these effects require a multi-faceted approach that targets the various dimensions of stress response, including water relations, membrane integrity, and antioxidant defence. This study evaluated the combined and individual effects of polyethylene glycol (PEG) as an osmotic primer and proline as an osmoprotectant on salinity-stressed mango trees. The study evaluated the effects of PEG (15 and 30 mM) and proline (8 and 13 mM) treatments on mango plants, with specific emphasis on their interactive responses under stress conditions over two consecutive growing seasons.

Relative water content (RWC), a key indicator of osmotic adjustment and turgor maintenance [49], significantly improved under combined PEG and proline treatments, particularly in T_6_ (PEG 30 mM + Proline 13 mM) and T_8_ (PEG 30 mM + Proline 8 mM), confirming that osmotic regulation under salinity is best supported by either stress signalling (via PEG) or osmoprotection (via proline). These findings align with the osmotic stress adaptation model proposed by Tester and Davenport [4] and Verslues et al. [22], confirming the role of PEG in triggering ABA-mediated stomatal regulation and aquaporin activation, thereby sustaining cellular hydration under salt stress. Parallel trends were observed in the membrane stability index (MSI), an indicator of resistance to oxidative membrane injury. T_6_ and T_8_ exhibited the highest MSI values, indicating enhanced membrane stabilization and reduced ion-induced leakage compared to both control and single-agent treatments. This outcome is consistent with previous findings reported by Lamlom et al. [50] and Rhaman et al. [51], which suggest that the combined application of glycine betaine and proline enhances lipid bilayer stability and reduces oxidative peroxidation. In this context, RWC correlated positively (*p <* 0.01*)* with MSI (*r* = 0.88 and 90 in the first and second season, respectively), confirming that water conservation reduces ionic leakage and oxidative membrane disruption [16, 32, 52].

The antioxidant defence system in mango trees responded markedly to PEG and proline treatments, with enhancement observed in enzymatic and non-enzymatic components. Although PEG-alone treatments also maintained elevated PPO activity, the magnitude was consistently lower than that observed in the combined treatments. Peroxidase (POD) and polyphenol oxidase (PPO) activities were significantly elevated in PEG-dominant treatments, especially T_6_ (PEG 30 mM + Proline 13 mM) and T_8_ (PEG 30 mM + Proline 8 mM), reflecting enhanced ROS-scavenging capacity [7–9, 53, 54]. A strong positive correlation between POD and PPO activities was observed, as indicated by Pearson’s correlation coefficients in both the first season (*r* = 0.90, *p <* 0.01) and the second season (*r* = 0.94, *p <* 0.001). These results align with the foundational work of Nimbolkar et al., [52], and Sairam et al. [55], who characterized these enzymes as critical components of redox homeostasis under abiotic stress. Moreover, polyphenol oxidase (PPO) activity exhibited a marked increase in treatments T_6_ and T_8_, aligning with the findings of Singh et al. [56] and Perveen et al. [57], who associated PPO activity with phenolic compound oxidation and cell wall reinforcement under salinity stress. The observed increase in PPO activity in T_8_ compared to the control (approximately 41%) underscores the synergistic role of PEG and proline in preserving enzymatic function under saline conditions. The enhanced activity of PPO in proline-enriched treatments reflects proline’s role not only in osmotic buffering but also in maintaining redox homeostasis [7, 21, 30, 52–54]. Moreover, these findings align with those of Elsheery et al. [13] and Othman [58], who reported that control mango trees exhibited values comparable to those observed in the present study, serving as a basis for comparison. Ascorbate, a key non-enzymatic antioxidant, exhibited pronounced accumulation in PEG-treated mango trees, particularly under T_6_ (PEG 30 mM + Proline 13 mM) and T_8_ (PEG 30 mM + Proline 8 mM), highlighting PEG’s role in priming antioxidant biosynthesis [33]. This upregulation is further supported by the synergistic influence of proline, which facilitates NADP⁺/NADPH cycling, thus sustaining redox balance under oxidative stress [21, 33, 59]. Notably, a decline in ascorbate content during Season 2 in proline-alone treatments suggests possible feedback inhibition or depletion under prolonged stress exposure, underscoring the need for dual-agent interventions for sustained protection. These observations confirm the concentration-dependent and complementary effects of PEG and proline, not only in enhancing antioxidant buffering but also in preserving pigment integrity and regulating phenolic metabolism. Collectively, the coordinated elevation of ascorbate under PEG–proline treatments reflects a critical component of the integrated stress defence strategy in mango under saline conditions.

Chlorophyll serves as a critical indicator of photosynthetic efficiency and pigment integrity, particularly under salinity-induced oxidative stress. In this study, treatments T_6_ (PEG 30 mM + Proline 13 mM) and T_8_ (PEG 30 mM + Proline 8 mM) consistently recorded the highest total chlorophyll (a + b) content across both growing seasons. This enhancement reflects improved chloroplast stability and photosynthetic function and aligns with the findings of Tsai et al. [60], who emphasized chlorophyll preservation as a proxy for stress tolerance and photosynthetic resilience. The elevated chlorophyll levels in T_6_ and T_8_ support the chloroplast protection model, wherein osmotic buffering by PEG and membrane stabilization via proline mitigate pigment degradation. In contrast, T_5_, which lacked optimal proline supplementation, exhibited significantly lower chlorophyll concentration, suggesting greater pigment breakdown under metabolic strain. Notably, control trees exposed to salinity stress without any protective treatment showed the most severe chlorophyll loss, underscoring the detrimental impact of salt stress on photosynthetic pigments and the protective role of combined osmolyte application. The strong positive correlation between chlorophyll content and yield (Season 1: *r* = 0.90, *p <* 0.001; Season 2: *r* = 0.81, *p <* 0.01) confirms that sustained photosynthetic efficiency directly enhances productivity in mango. The consistency of this relationship across both growing seasons reinforces chlorophyll concentration as a reliable physiological indicator of yield potential under stress conditions. Moreover, the photosynthetic efficiency, indicated by total chlorophyll and leaf area, responded positively to combined PEG–proline treatments. These benefits likely reflect the stabilization of chloroplast membranes and pigment-protein complexes under combined osmotic and antioxidant support [11, 16, 27, 61]. Furthermore, salinity stress has been reported to reduce the number of chloroplasts and cause structural damage to thylakoid and plastid membranes due to membrane decomposition. Additionally, the degradation of chloroplasts may stimulate the activity of chlorophyllase, thereby contributing to a reduction in chlorophyll content [52, 62, 63].

Total soluble sugars (TSS) increased significantly across all PEG and proline treatments compared to the control, which consistently recorded the lowest values. The highest TSS levels were also observed in T_6_ (PEG 30 mM + Proline 13 mM) and T_8_ (PEG 30 mM + Proline 8 mM), indicating a strong synergistic effect between PEG and proline in enhancing carbohydrate metabolism and osmotic buffering. These findings align with Ali et al. [64], who highlighted the role of sugar accumulation as a critical component of osmotic adjustment and metabolic resilience under abiotic stress. While other treatments, including PEG or proline alone, showed elevated sugar levels, they did not match the sustained enhancement seen in the combined treatments. The proline-driven increase in TSS likely contributes to energy buffering and the stabilization of cellular structures, ensuring continued metabolic function under prolonged salt stress. Thus, the data reinforces the concentration- and interaction-dependent impact of PEG and proline on sugar-mediated osmoprotection in mango trees exposed to salinity.

Proline functions as a key osmoprotectant, contributing to protein and membrane stabilization, reactive oxygen species (ROS) detoxification, and maintenance of osmotic and redox homeostasis [18, 19, 21, 30, 52, 65]. Its accumulation also correlates with the activation of stress-responsive genes, including those encoding (P5CS), under regulation by abscisic acid (ABA) and oxidative cues [26, 66]. PEG-induced osmotic stress activates ABA-dependent pathways, upregulating P5CS1 (the stress-responsive isoform) to enhance proline synthesis, as demonstrated in drought-tolerant species [67, 68]. Proline, in turn, stabilizes cellular redox balance, indirectly modulating ion transporters by mitigating oxidative stress [69, 70]. Notably, P5CS2 plays a developmental role but may contribute to ion homeostasis under prolonged stress [71]. In contrast control trees showed significantly lower internal proline concentrations. Notably, proline levels in T_6_ (PEG 30 mM + Proline 13 mM) and T_8_ (PEG 30 mM + Proline 8 mM) increased in both seasons, indicating a possible priming or metabolic memory effect linked to ABA-proline feedback loops and epigenetic regulation [72]. These findings collectively demonstrate that T_6_ (PEG 30 mM + Proline 13 mM) and T_8_ (PEG 30 mM + Proline 8 mM) are more effective at sustaining high internal proline content, thereby reinforcing cellular hydration, antioxidant capacity, and adaptive stress tolerance mechanisms in mango under salinity.

Hormonal profiling revealed that treatments T_6_ (PEG 30 mM + Proline 13 mM) and T_8_ (PEG 30 mM + Proline 8 mM) consistently exhibited the highest IAA/ABA ratios across both seasons, supporting the hypothesis that PEG enhances auxin signalling while modulating ABA-mediated stress responses [41, 73]. These elevated ratios suggest a shift toward growth-promoting hormonal balance, enabling continued shoot elongation, flush emergence, and reproductive development under salinity stress [14, 16, 74]. While proline-alone treatments maintained moderate IAA/ABA ratios, their sustained effect appears dependent on PEG-initiated rebalancing. The combined treatments not only fostered hormonal equilibrium but also improved metabolic stability through osmotic regulation, reflecting a synergistic interaction between PEG and proline. This synergy translated into significant agronomic improvements, as evidenced by the superior total yield observed in treatments T_6_ and T_8_, reflecting more efficient assimilate distribution and enhanced capacity for photoassimilate utilization, consistent with the overview of Wahid et al. [75]. In contrast, control trees exhibited significantly lower IAA/ABA ratios and corresponding reductions in vegetative and reproductive performance, emphasizing the necessity of osmotic and hormonal intervention for maintaining growth and productivity under saline conditions. Although both proline and PEG contribute to plant responses under salinity stress and have been linked to alterations in phytohormone levels, direct evidence of their specific effects on cytokinins or ethylene remains limited. It is well established that salinity stress disrupts hormonal homeostasis, often leading to significant changes in levels of abscisic acid, cytokinins, ethylene, and auxins, which collectively influence plant growth and stress adaptation [76, 77].

Consequently, the observed physiological and biochemical responses attributable to the synergistic effect of PEG and proline were accompanied by significant improvements in agronomic traits. In contrast, untreated trees exhibited the lowest shoot length across both seasons, highlighting the combined role of osmotic stimulation and osmoprotective buffering, as reported by Hayat et al. [27]. The number of flushes, a key yield precursor in mango, was highest in T_6_ (PEG 30 mM + Proline 13 mM) and T_8_ (PEG 30 mM + Proline 8 mM), confirming that PEG and combined treatments promote meristematic reactivation and auxin-related growth cycles [21]. Also, the leaf area was larger in T_6_ and T_8_ across both seasons, supporting the findings of Williams and Araujo [78], who emphasized the importance of canopy surface preservation under salt stress. Abd Elsamad et al. [16] and Mahouachi et al. [79], working on mango trees under salinity stress, reported a similar trend, where leaf area was markedly reduced in untreated trees exposed to abiotic stress. This aligns with the current findings, underscoring the impact of stress mitigation strategies on maintaining vegetative growth under adverse conditions. In this context, PEG priming likely enhanced turgor and IAA transport, while proline helped maintain membrane fluidity and chloroplast integrity. Overall, these trends reinforce that PEG, especially in combination with proline, promotes vegetative vigour and canopy development under prolonged saline stress.

Building upon the observed improvements in vegetative traits under combined osmotic and osmoprotective treatments, the reproductive responses further illustrate the functional synergy between PEG and proline in sustaining productivity under salinity stress. Fruit set percentage peaked in T_6_ and T_8_, likely facilitating fertilization via osmotic balance and enhancing pollen viability and stigma receptivity [80, 81]. Also, these findings are consistent with prior work showing the importance of carbohydrate availability and hormonal balance for fruit retention [21]. Fruit weight was highest in T_9_ (control), affirming that combined PEG–proline treatment enhances sink strength and metabolic loading under stress, as noted by El-Tayeb [44] and Wahid et al. [75]. It is certain that the significant increase in fruit weight may be due to the small number of fruits on the tree, which reduces competition between fruits for internal nutrients. Yield was significantly and positively correlated with all ascorbate (*r* = 0.91, *p <* 0.001), flushes number (*r* = 0.95, *p <* 0.001), total chlorophyll (*r* = 0.90, *p <* 0.001), TSS (*r* = 0.93, *p <* 0.001), fruit set (*r* = 0.95, *p <* 0.001), RWC (*r* = 0.91, *p <* 0.001), shoot length (*r* = 0.96, *p <* 0.001), PPO (*r* = 0.89, *p <* 0.01), POD (*r* = 0.86, *p <* 0.01) and leaf area (*r* = 0.88, *p* < 0.01) in the first season. The observed strong positive correlations among relative water content (RWC), shoot elongation, polyphenol oxidase (PPO) activity, and yield emphasize the interconnected roles of osmotic regulation and oxidative stress responses in maintaining mango productivity under saline conditions. Elevated RWC appears to support cellular turgor pressure, which in turn promotes shoot growth and canopy expansion. These processes are essential for preserving photosynthetic efficiency during salinity-induced stress. At the same time, increased PPO activity indicates an upregulation of phenolic metabolism, which may contribute to the stabilization of cell walls and the reduction of oxidative damage. The integration of these physiological traits likely contributes to the stability of yield under salinity stress. Sustained shoot growth and elevated relative water content may enhance water-use efficiency, thereby supporting fruit set and retention during periods of stress. Additionally, stress acclimation mediated by polyphenol oxidase activity may help mitigate oxidative damage, reducing the likelihood of premature fruit abscission. Similar results were found in the second season. Total yield per tree aligned with these reproductive trends, peaking in T_6_ and T_8_ significantly in respective seasons. This confirms that while PEG may enhance early season output, proline plays a crucial role in maintaining productivity under prolonged stress conditions [44]. The consistent negative correlations between fruit weight and key physiological traits across both seasons, particularly MSI, RWC, and total yield, indicate a physiological trade-off rather than a methodological artifact. These significant negative correlations imply that higher fruit weight may come at the expense of other physiological and yield-related processes, possibly due to resource allocation trade-offs or compensatory metabolic shifts. This pattern aligns with established theories of assimilate partitioning in fruiting plants, where resources are allocated to fruit development at the expense of vegetative growth, such as reduced shoot length, and reduced investment in stress defence mechanisms, including lower PPO activity. The observed inverse relationships with osmoprotectants such as proline and TSS, as well as with chlorophyll content, further suggest that greater reproductive investment may limit the metabolic capacity required for effective stress response. Notably, the lack of a significant correlation with the IAA to ABA ratio suggests that this shift in resource allocation may occur independently of major phytohormonal control. These findings support the interpretation of an adaptive compromise, in which increased fruit weight reflects successful reproductive prioritization but may also be associated with reduced physiological resilience and secondary metabolic activity.

It is noteworthy that the first four treatments (T_1_, T_2_, T_3_, and T_4_), which involved the individual application of either PEG or proline, exhibited similar results and followed a consistent trend across all measured characteristics. However, their effects were less pronounced compared to the combined treatments. Nonetheless, each of these treatments demonstrated noticeable improvements over the control, indicating that even individual applications of PEG or proline contributed positively to mitigating salinity stress. Furthermore, PEG is known to simulate drought-like osmotic stress, which effectively triggers the plant’s internal stress signalling mechanisms, including enhanced abscisic acid (ABA) accumulation. This hormonal response promotes efficient stomatal closure, thereby reducing transpirational water loss. In contrast, proline functions primarily as a compatible solute and metabolic regulator that supports osmotic balance and stress tolerance but does not directly activate ABA signalling to the same extent [82–84]. Therefore, PEG alone appears to be more effective than proline alone in priming ABA-mediated stomatal closure, due to its ability to activate intrinsic drought response pathways. The data suggests that yield optimization under salinity is best achieved when early-stage osmotic signalling (via PEG) is sustained by metabolic support (via proline) through the full reproductive cycle. Under salinity stress, mango and other plant species reprogram their metabolic pathways to accumulate osmoprotective amino acids such as proline, glutamine, and arginine, while reducing levels of precursors like glutamate, thereby supporting cellular homeostasis and providing additional protective functions beyond the role of proline alone [85]. Furthermore, salt stress alters carbohydrate metabolism, leading to the accumulation of glucose and glucose-6-phosphate, which are key intermediates that contribute to energy balance and cellular repair processes. These metabolic adjustments also promote the synthesis of phenolic compounds, flavonoids, and other antioxidant molecules, thus enhancing the plant’s defence against salinity-induced oxidative stress [86]. In addition, the exogenous application of proline has been shown to upregulate genes involved in its own biosynthesis, such as P5CS (Δ¹-pyrroline-5-carboxylate synthase) and P5CR (Δ¹-pyrroline-5-carboxylate reductase), as well as genes encoding antioxidant enzymes like superoxide dismutase (SOD), ascorbate peroxidase (APX), and catalase (CAT), which collectively enhance the detoxification of reactive oxygen species (ROS) under saline conditions [76]. Moreover, salinity and proline treatments influence the expression of genes related to nutrient uptake and ion transport, including aquaporins and sodium transporters, thereby improving the plant’s capacity to maintain ionic homeostasis and reduce ion toxicity.

**Fig. 11:**
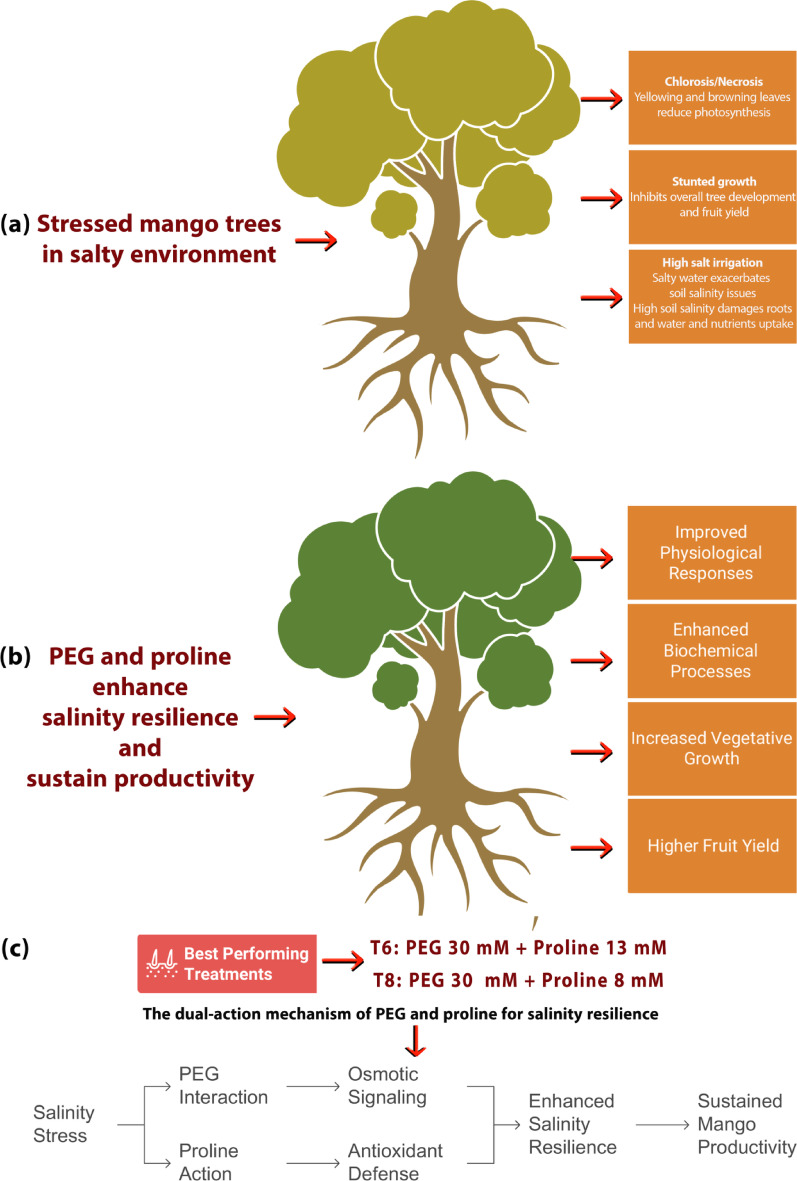
Graphical representation illustrates the dual protective effects of polyethylene glycol (PEG) and proline in enhancing salinity resilience in mango (*Mangifera indica*) trees. (**a**) Mango trees under salinity stress showing visible symptoms and annotated physiological effects. (**b**) Mitigating effects of PEG and proline, highlighting improved plant condition. (**c**) Key treatment combinations and a model illustrating their synergistic mechanism for salinity resilience

Hormonal signalling pathways are also affected, particularly those involving abscisic acid (ABA) and auxin, which regulate stomatal behaviour, root growth, and other adaptive responses essential for stress tolerance [87]. Finally, proline acts as a molecular chaperone by stabilizing proteins and cellular membranes against denaturation, and its accumulation is often accompanied by an increase in compatible solutes and stress-responsive proteins that reinforce cellular structures and enhance resilience to salt-induced damage [88].

Taken together, these findings validate the integrative model wherein PEG primes the plant’s osmotic and hormonal responses, while proline supplements membrane protection and antioxidant resilience. The combined application of both compounds, especially at optimized levels in T_6_ (PEG 30 mM + Proline 13 mM) and T_8_ (PEG 30 mM + Proline 8 mM), enables mango trees to withstand salinity-induced physiological and biochemical disruptions as illustrated in Fig. 11. This interaction-dependent strategy thus offers a promising agronomic approach for enhancing salinity tolerance in mango cultivation. Practically, applying proline and polyethylene glycol (PEG) in combination, especially at the optimized concentrations found in treatments T_6_ (PEG 30 mM with Proline 13 mM) and T_8_ (PEG 30 mM with Proline 8 mM), provides an effective strategy for improving salinity tolerance in mango.

## Conclusion

Importantly, this is the first study to report the synergistic effects of PEG and proline co-application in a perennial fruit crop under saline conditions, offering practical implications for sustainable orchard management and new directions for stress physiology research. This study revealed that salinity stress triggers multifaceted physiological disturbances in mature mango trees, manifesting as osmotic dehydration, membrane instability, oxidative damage, and hormonal dysregulation. Practically, the combined application of PEG as an osmotic agent and proline as an osmoprotectant, particularly at optimized levels such as in T_6_ (PEG 30 mM + proline 13 mM) and T_8_ (PEG 30 mM + proline 8 mM), offers an effective strategy to enhance salinity tolerance in mango. This combination effectively mitigates stress responses and can be applied through foliar or soil methods during critical developmental stages, especially flowering and fruit set, in salt-affected orchards. These combinations enhanced multiple physiological parameters including RWC, MSI, antioxidant activity (POD, PPO, and ascorbate), photosynthetic pigment preservation, TSS, hormonal balance, vegetative growth (shoot elongation, leaf expansion, flush production), and reproductive performance (fruit set and yield). The findings propose a dual-mechanism model in which PEG initiates early stress signalling while proline maintains long-term physiological protection, offering a practical approach for sustaining mango productivity in saline environments. Specifically, PEG triggers osmotic alertness by activating ABA signalling and moderate ROS production, whereas proline sustains physiological function by stabilizing cellular structures and supporting redox and hormonal resilience. When properly dosed, their combined application provides multi-layered protection that not only mitigates damage but also optimizes adaptive responses under chronic salinity stress. While this study highlights the physiological benefits of PEG and proline, it has some limitations. The underlying molecular mechanisms (e.g., stress-related gene expression or proteomic changes) were not explored, potential long-term effects of PEG accumulation in orchard soils remain unclear, and findings are limited to the ‘Ewais’ cultivar under Egyptian conditions. These points emphasize the need for molecular validation, extended field studies, and multi-location trials to assess broader applicability.

### Future perspectives

The proven synergy between polyethylene glycol (PEG) and proline in mango trees under salt stress opens new opportunities for integrated stress management. Future research should adopt a comprehensive approach to optimizing salinity management in mango. Priorities include identifying the molecular basis of PEG- and proline-induced stress tolerance through transcriptomic and metabolomic profiling, evaluating a broader range of application concentrations, and investigating interactions with diverse rootstocks. Direct assessments of photosynthetic performance, such as gas exchange and chlorophyll fluorescence, are also recommended to clarify physiological responses. Additional strategies may involve combining PEG with ascorbic acid to boost antioxidant defences, with nano-formulations to enhance uptake, with plant growth regulators to improve resilience, and with silicon to support ionic balance. Multi-season field trials assessing yield and fruit quality, along with economic feasibility and omics-based analyses, will be essential for developing scalable, genotype-specific solutions for mango cultivation under saline and water-limited conditions.

## Supplementary Information

Below is the link to the electronic supplementary material.

Supplementary Material 1

Supplementary Material 2

Supplementary Material 3

## Data Availability

The data generated during and/or analysed during the current study are available from the corresponding author on reasonable request.
