# Supplementary material for: Polyethylene glycol and proline synergistically improve salinity tolerance via physiological and biochemical reprogramming in mango
Source: BMC Plant Biol. 2025 Aug 29;25:1161. doi: 10.1186/s12870-025-07211-4 (PMC12395829; doi:10.1186/s12870-025-07211-4)
Supplement: Supplementary file 1 — Supplementary Material 1 [file 12870_2025_7211_MOESM1_ESM.docx]

# Soil Analysis – Private Orchard

This document presents specific soil data collected from a scientific study conducted in Wadi El Natrun, Beheira Governorate, Egypt. The data reflects constant values measured directly from the field, providing a clear representation of soil conditions relevant to salt stress and crop tolerance.

| Property | Measured Value | Details |
| --- | --- | --- |
| Texture (0–15 cm depth) | Loamy sand (LS) | 58% medium sand, 12% fine sand, 5.7% clay |
| pH (paste extract) | 7.98 | Mildly alkaline |
| EC (saturated paste) | 1.23 dS/m | Moderate salinity level |
| SAR | 3.65 | Sodicity risk exists but not extreme |

## Interpretation & Implications

• Texture: Indicates well-drained conditions but with low nutrient and water retention.
• pH ≈ 7.98: Slightly alkaline; may restrict micronutrient uptake such as iron and zinc.
• EC ≈ 1.23 dS/m: Low to moderate salinity; acceptable for many crops.
• SAR ≈ 3.65: Sodicity may affect soil structure over time if unmanaged.
