# Supplementary material for: Polyethylene glycol and proline synergistically improve salinity tolerance via physiological and biochemical reprogramming in mango
Source: BMC Plant Biol. 2025 Aug 29;25:1161. doi: 10.1186/s12870-025-07211-4 (PMC12395829; doi:10.1186/s12870-025-07211-4)
Supplement: Supplementary file 2 — Supplementary Material 2 [file 12870_2025_7211_MOESM2_ESM.docx]

**Wadi El Natrun region, Egypt - Monthly climate summary**

| **Month 2023** | **Avg High (°C)** | **Avg Low (°C)** | **Mean Temp (°C)** | **Avg Rainfall (mm)** |
| --- | --- | --- | --- | --- |
| **January** | 23 | 10 | 16 | 0.2 |
| **February** | 26 | 11 | 18 | 0.1 |
| **March** | 30 | 15 | 23 | 0.9 |
| **April** | 35 | 20 | 28 | 0.7 |
| **May** | 40 | 25 | 32 | 0.4 |
| **June** | 42 | 27 | 34 | 0.1 |
| **July** | 42 | 28 | 35 | 0.2 |
| **August** | 42 | 28 | 35 | 0.0 |
| **September** | 40 | 26 | 33 | 0.2 |
| **October** | 37 | 22 | 29 | 0.7 |
| **November** | 30 | 16 | 23 | 0.1 |
| **December** | 25 | 11 | 18 | 0.0 |

| **Month 2024** | **Avg High (°C)** | **Avg Low (°C)** | **Mean Temp (°C)** | **Avg Rainfall (mm)** |
| --- | --- | --- | --- | --- |
| **January** | 23 | 10 | 16 | 5 |
| **February** | 26 | 11 | 18 | 10 |
| **March** | 30 | 15 | 23 | 15 |
| **April** | 35 | 20 | 28 | 10 |
| **May** | 40 | 25 | 32 | 5 |
| **June** | 42 | 27 | 34 | 0 |
| **July** | 42 | 28 | 35 | 0 |
| **August** | 42 | 28 | 35 | 0 |
| **September** | 40 | 26 | 33 | 0 |
| **October** | 37 | 22 | 29 | 5 |
| **November** | 30 | 16 | 23 | 10 |
| **December** | 25 | 11 | 18 | 15 |

Estimated using climatological data from Egypt Tours Portal and World Bank Climate Knowledge Portal. Egypt Tours Portal. (2023 and 2024). Climate and Attractions of Wadi El Natrun. Egypt Tours Portal, Egypt Travel Blog.
