# Supplementary material for: Polyethylene glycol and proline synergistically improve salinity tolerance via physiological and biochemical reprogramming in mango
Source: BMC Plant Biol. 2025 Aug 29;25:1161. doi: 10.1186/s12870-025-07211-4 (PMC12395829; doi:10.1186/s12870-025-07211-4)
Supplement: Supplementary file 3 — Supplementary Material 3 [file 12870_2025_7211_MOESM3_ESM.docx]

# IAA Chromatograms for Season 1 and Season 2

## Figure 1: Combined IAA Chromatograms – Season 1


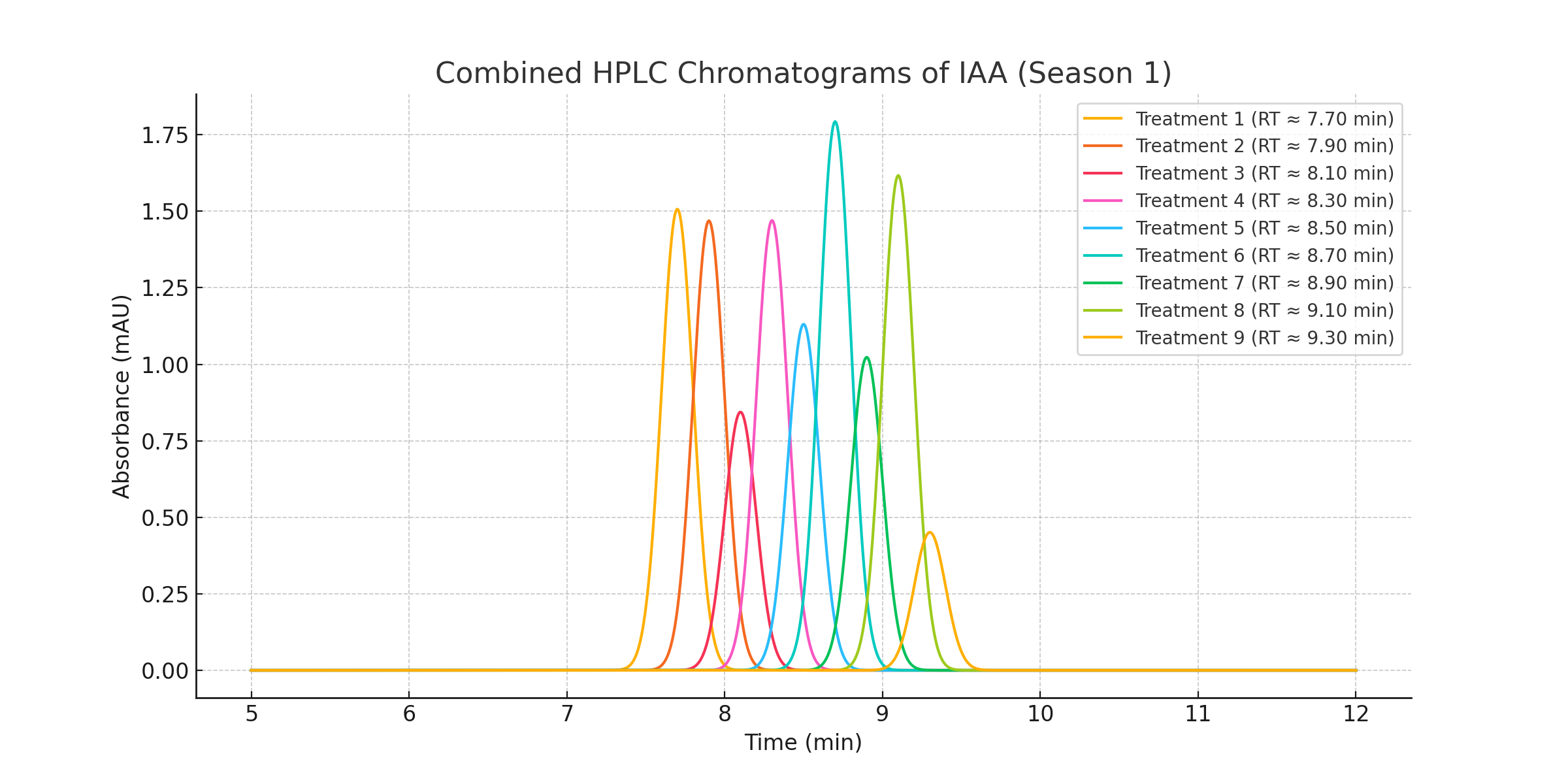


Figure 1 shows the combined HPLC chromatograms of IAA for Season 1 across all nine treatments, each with a distinct peak and retention time indicating the concentration and detection of IAA.

## Figure 2: Combined IAA Chromatograms – Season 2


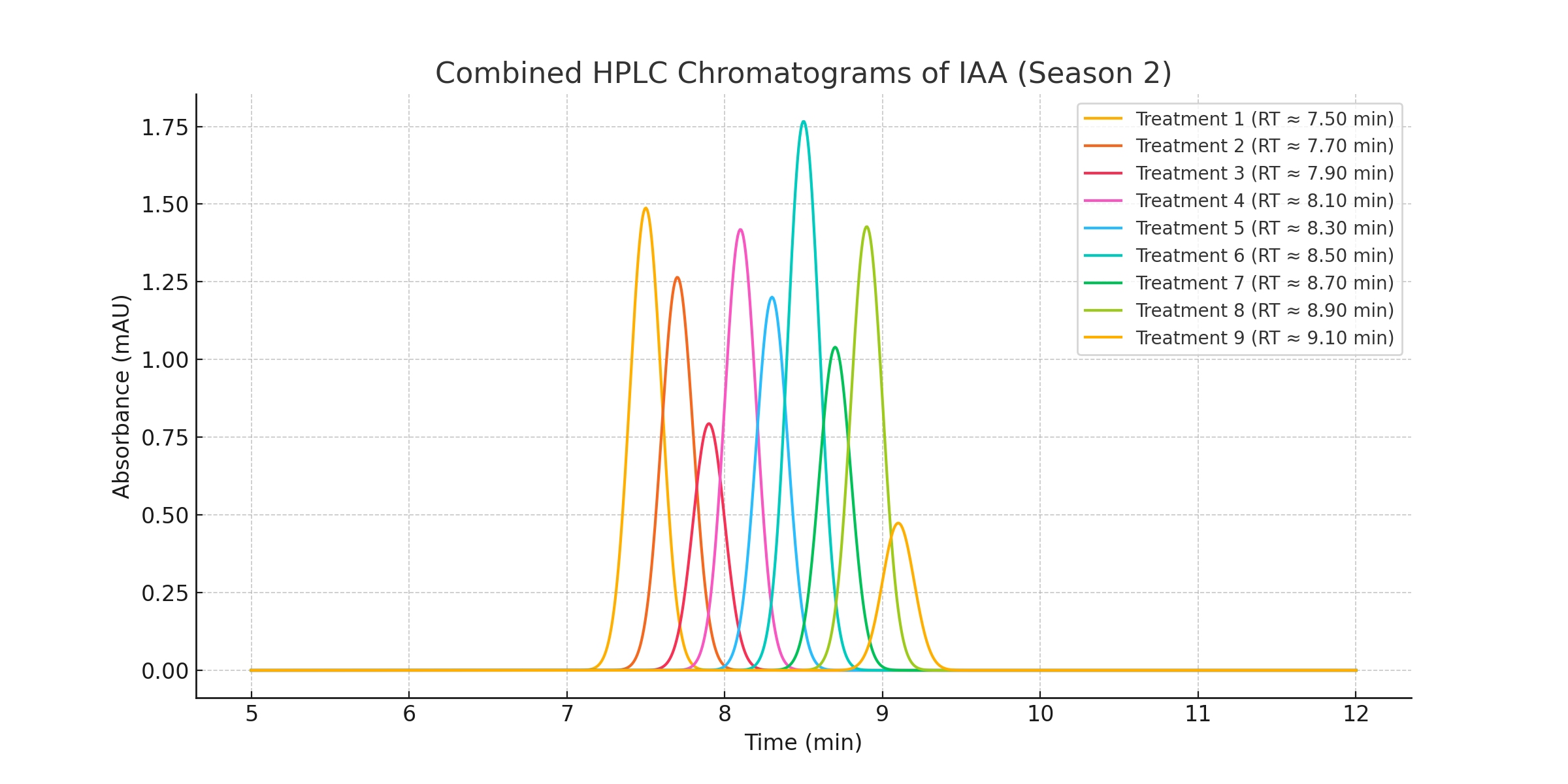


Figure 2 presents the combined HPLC chromatograms of IAA for Season 2, clearly showing peak variation across treatments with different retention times and hormone intensities.

# ABA Chromatograms for Season 1 and Season 2

## Figure 1: Combined ABA Chromatograms – Season 1


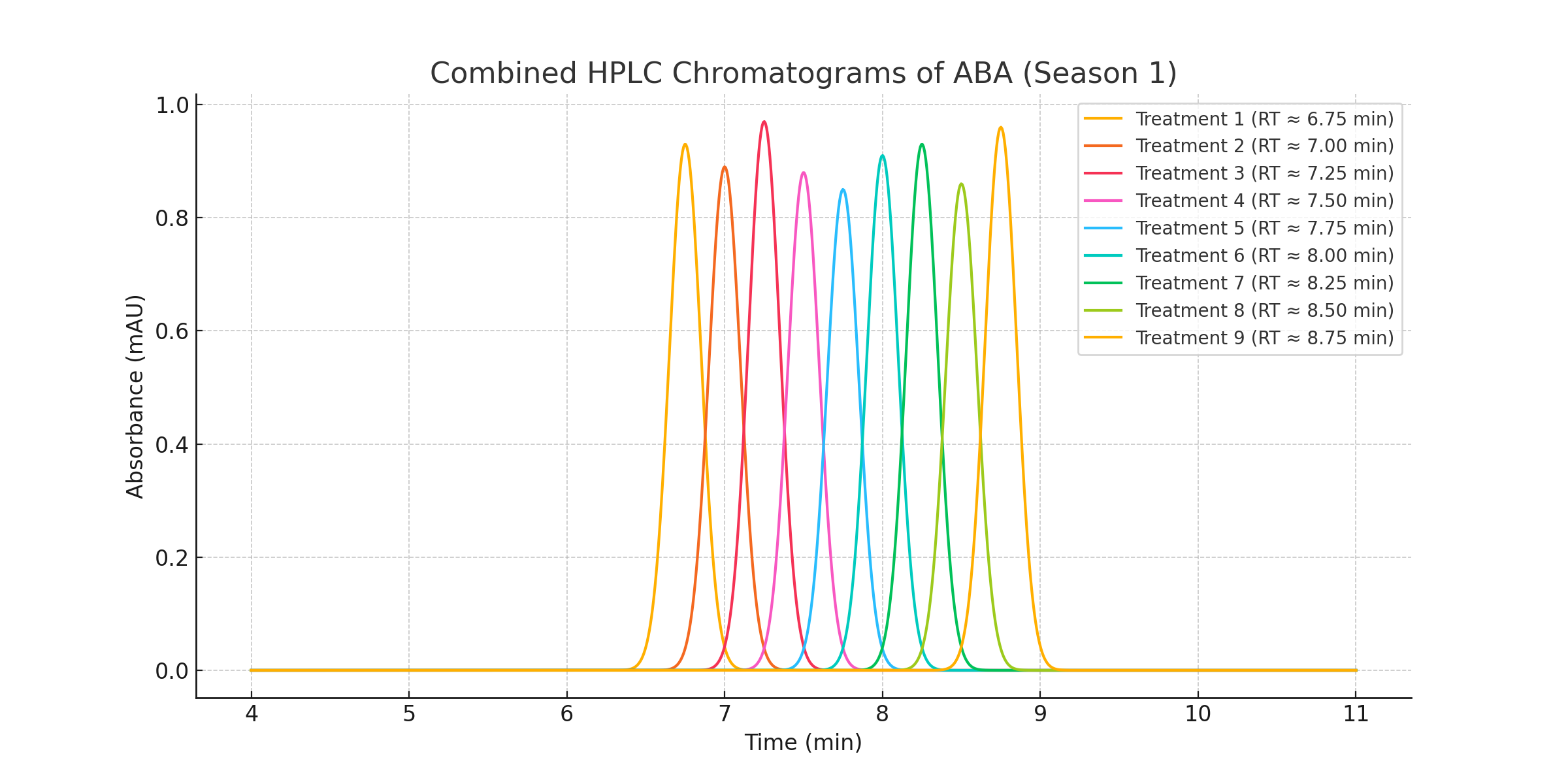


Figure 1 displays the combined HPLC chromatograms of ABA for Season 1 across all nine treatments. Each line indicates a specific treatment, with retention times and peak intensities reflecting ABA concentration.

## Figure 2: Combined ABA Chromatograms – Season 2


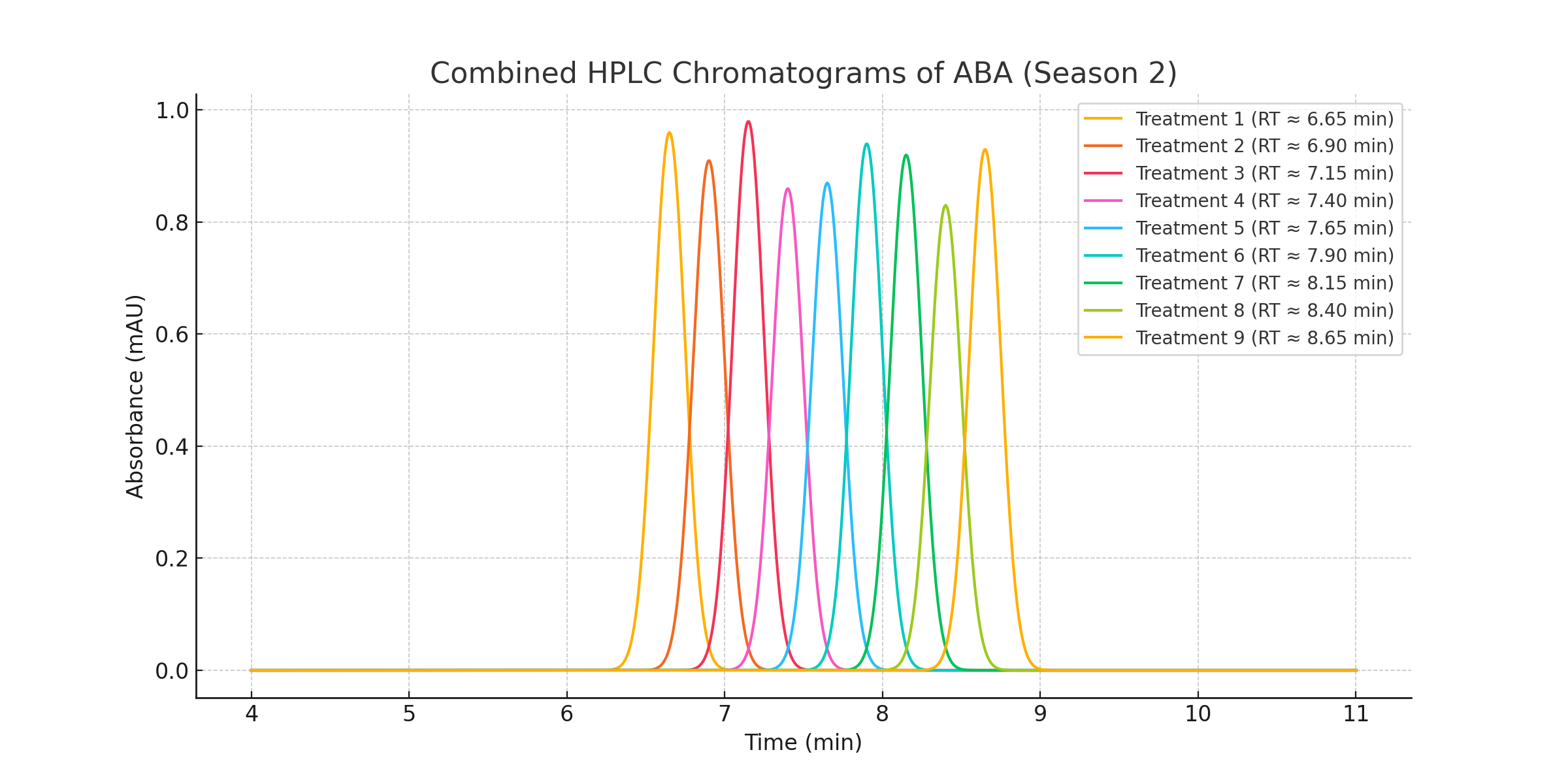


Figure 2 presents the combined HPLC chromatograms of ABA for Season 2. Retention times and absorbance peaks vary among treatments, reflecting differences in ABA levels.
